# Kinetic Analysis of the Redox-Neutral Catalytic Mitsunobu Reaction: Dehydration, Kinetic Barriers, and Hopping between Potential Energy Surfaces

**DOI:** 10.1021/jacs.5c05404

**Published:** 2025-05-13

**Authors:** Keith G. Andrews, Stefan Borsley

**Affiliations:** Department of Chemistry, 3057 Durham University, Lower Mount Joy, South Road, Durham DH1 3LE, U.K.

## Abstract

Denton’s redox-neutral catalytic Mitsunobu reaction is remarkable in that it translates a reaction traditionally driven by the consumption of sacrificial chemical reagents to an additive-free catalytic manifold. Rational attempts to improve the system have been met with only marginal improvements, and a lack of consensus concerning the rate-determining step continues to limit effective reaction development. Here, we analyze the reaction mechanism focusing on a critical, largely overlooked element: the removal of water using a Dean–Stark apparatus. Experimental analysis of the water removal process, coupled with extensive kinetic simulations, demonstrates that the overall rate of the reaction is intimately tied to the rate of water removal. This process can be viewed as a transition between potential energy surfaces and, consequently, subsequent steps of the reaction can progress spontaneously in the absence of water, allowing an explanation of how Le Chatelier’s principle, a thermodynamic effect, can have a profound kinetic influence over the rate of the reaction. We identify three bottlenecks in the reaction that inform catalyst design. Additionally, we (a) clarify the ongoing discussion regarding the rate-determining step, (b) provide clear advice concerning future reaction design taking into account the role of water and, (c) discuss the redox-neutral catalytic Mitsunobu reaction in the context of formally endergonic esterification reactions, noting parallels with ratchet mechanisms. Finally, we highlight general principles of catalyst/reaction design that emerge from our analysis and implement our findings to demonstrate a 50% rate acceleration resulting from improved water removal, a substantially greater reaction enhancement than previously obtained from computationally guided catalyst structural changes.

## Graphical abstract

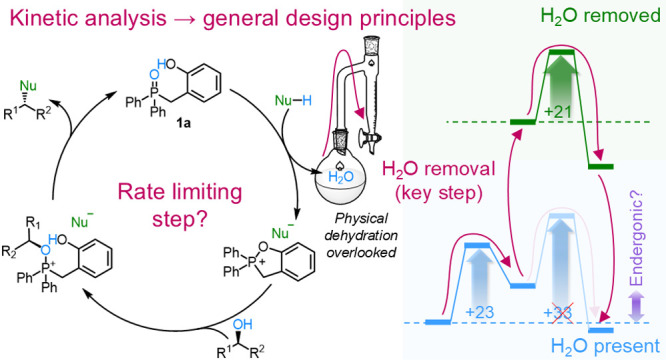

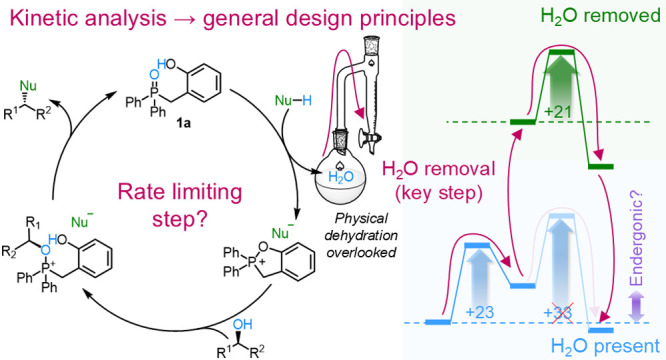

## Introduction

The Mitsunobu reaction is widely used to perform the stereoinversion of optically active alcohols via substitution by an acidic pronucleophile (Figure 1A).^1–4^ To overcome the high kinetic barrier of this reaction, the original Mitsunobu protocol^5–9^ exploited a highly reactive phosphine/azodicarboxylate reagent pair to drive the reaction, often at ambient temperatures and within a few hours.^10,11^ The reaction is a “redox dehydration”^12^ and a significant component of the thermodynamic and kinetic driving force is afforded by transfer of a molecule of water from the substrates to phosphine oxide and hydrazine waste products.^13^

**Figure 1:**
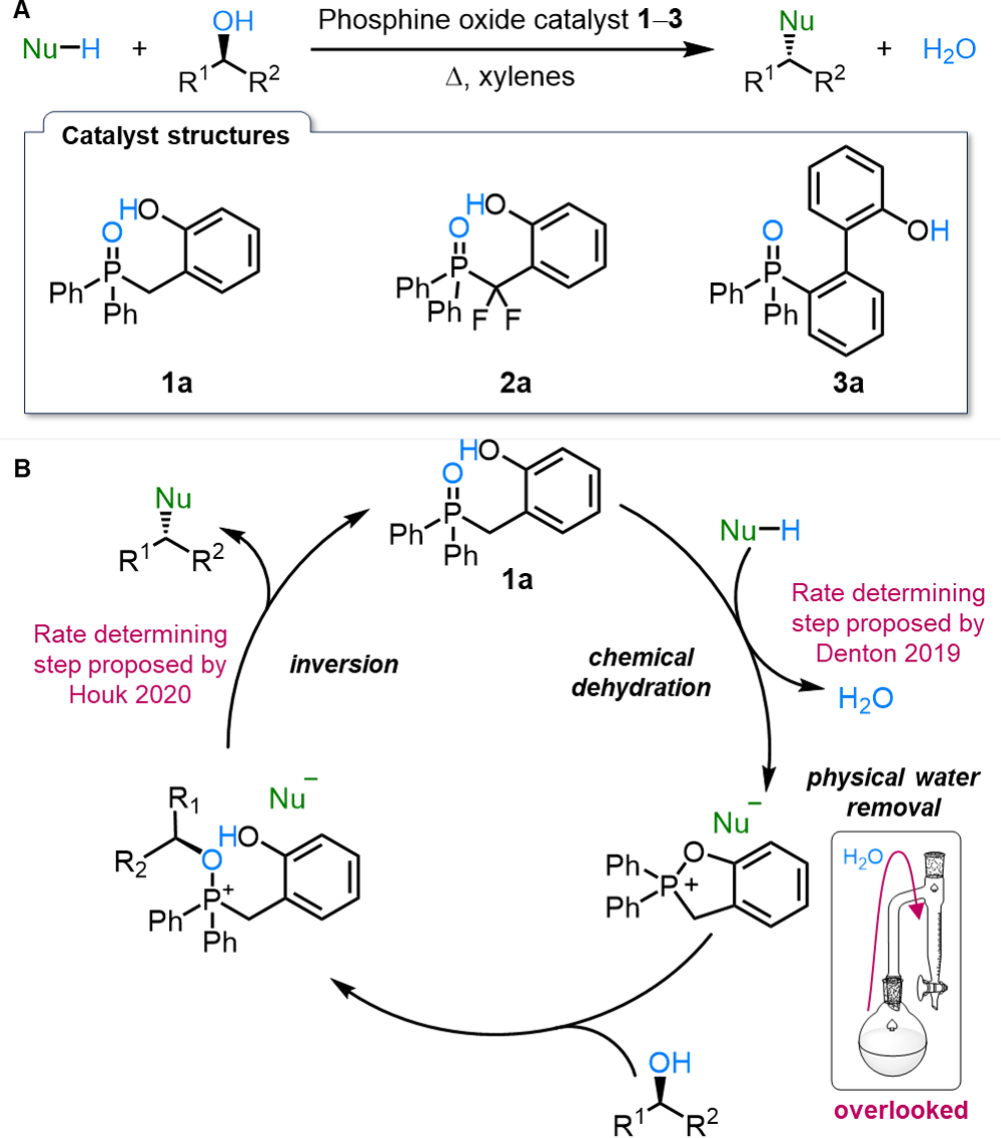
(A) Denton’s redox-neutral catalytic Mitsunobu reaction. (B) Denton’s proposed catalytic cycle.^14^

In 2019, one of us, working with Denton and co-workers, reported phosphine oxide catalyst **1a**,^14^ which performs the same Mitsunobu-type reaction without the need for additional stoichiometric reagents or redox-cycling, generating water as the only byproduct (Figure 1). This redox-neutral approach,^15–21^ unusual for *P*-catalytic manifolds,^22–30^ contrasts to previous catalytic Mitsunobu protocols,^21,31^ which use sacrificial redox reagents to recycle the phosphine oxide and/or hydrazine waste product(s) to the phosphine and/or azo oxidant.^32–36^ Other advances have been reported.^37–40^ In their proposed catalytic cycle (Figure 1B), Denton and co-workers highlighted two key features that drive catalysis. First, an internal phenol nucleophile accelerates *chemical dehydration* of the phosphine oxide (protonated in situ by the acidic pronucleophile), forming an activated phosphonium salt **1c**. Second, is the *physical removal of water* using azeotropic distillation (from homogeneous solution in toluene or xylenes) to a Dean–Stark trap,^42^ which selectively removes water due to its higher density. The authors described water removal as “critical···because···the phosphonium salt intermediates are···unstable with respect to hydrolysis” and so thought it likely that dehydration was turnover-limiting.^14^

Subsequently, Houk and co-workers employed density functional theory (DFT) calculations to appraise Denton’s catalytic cycle, reporting energy minima and transition state barriers under implicit toluene solvation (Figure 2A,B). Houk’s DFT analysis indicates that the highest free energy barrier in the system is the final C–O bond formation with inversion (nucleophilic coupling). On this basis, Houk and co-workers proposed a fluorinated catalyst (**2a**) designed to reduce the barrier of the nucleophilic coupling step, equivalent to a ∼1000-fold overall reaction rate increase.^41^

**Figure 2:**
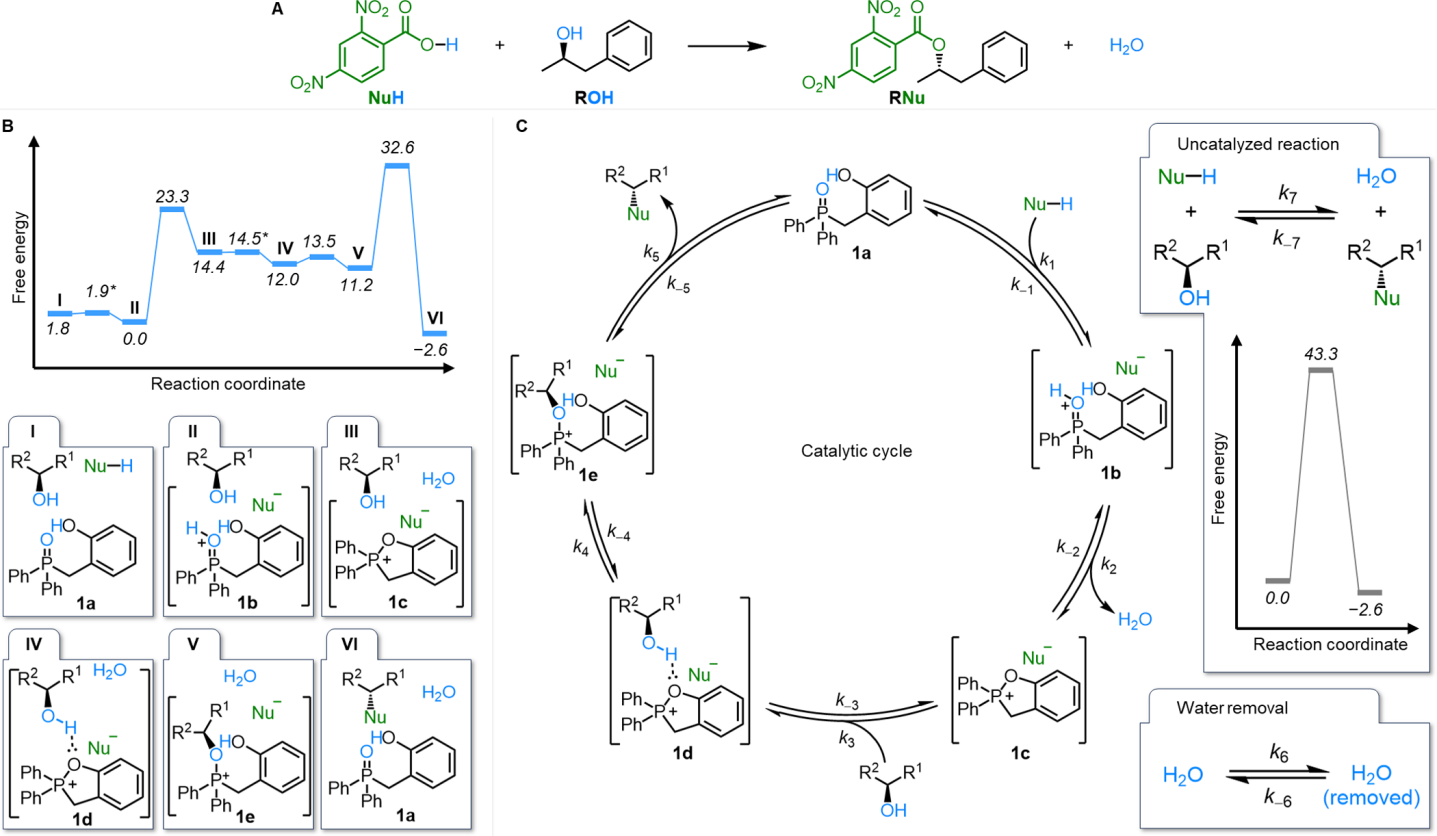
(A) Structures of reagents employed by Houk and co-workers^41^ for computational analysis of Denton’s catalytic Mitsunobu reaction.^14^ (B) Simplified scale diagram of the computational energy surface for the catalytic Mitsunobu reaction described by Houk and co-workers.^41^ Energies for all species and transition states are quoted in kcal mol^–1^. Where no computational barrier is provided, the process is assumed to be very fast, and an arbitrary barrier of 0.1 kcal mol^–1^ has been assumed for the forward process, and is indicated with an asterisk (*). (C) Reaction scheme showing the equilibria involved in the catalytic cycle, the uncatalyzed reaction, and the physical removal of water, that together describe the catalytic Mitsunobu reaction. The rate constants for the forward (*k*_n_) and backward (*k*_–n_) processes can be approximated from the calculated energy levels using the Eyring equation (see Supporting Information Section S2 for details).

Recently, Ling, Zhong and co-workers reported unsuccessful attempts at synthesizing hypothetical catalyst **2a**, so instead employed Houk’s DFT analysis to design phosphine oxide **3a** (Figure 1A).^43^ Despite presenting analogous calculations predicting a reduction in the energy barrier for the final nucleophilic coupling step using **3a**, only a marginal experimental rate and yield improvement (∼13%) was observed relative to Denton’s original catalyst (**1a**). Further, a separate kinetic study found partial (less than 1), positive orders of reaction for catalyst, pronucleophile and alcohol, suggesting a role for alcohol in the rate-limiting step, but also hinting that nucleophilic coupling is not uniquely turnover limiting.^44^ Since no intermediates have been observed in practice, experimentally determined microscopic rate constants remain unavailable. To expand the relevance of the catalytic Mitsunobu reaction (e.g., to industrially relevant reactions),^45–49^ it is necessary to clarify the rate-limiting step(s) of this reaction in order to facilitate the design of improved catalysts^41,43,44^ and conditions^50^ for this powerful transformation.

Here, we present an analysis of the catalytic Mitsunobu reaction that reconciles the apparent disagreement between the experimentally observed results and the computational predictions (Figure 1). We have used the computational data previously reported by Houk and co-workers to construct a kinetic model to describe the reaction (Figure 2). Our kinetic simulations, supported by experimental water removal measurements, reveal the crucial role of physical water removal from the system. Based on our findings, we present a revised description of the reaction in which the removal (and reintroduction) of water is represented through kinetic barrier diagrams or as a transition between different potential energy surfaces, allowing us to fully describe the driving principles of the reaction, and identify where improvements should be sought. Finally, we examine the implications of our analysis, discussing how the system might drive thermodynamically unfavored endergonic reactions,^51–53^ highlighting the parallels with ratchet mechanisms,^52,54^ and codifying how fundamental concepts from nonequilibrium chemistry^51,52,54^ might be applied in modern reaction design.

## Results and Discussion

### Constructing a Kinetic Model

The computational analysis of Houk and co-workers^41^ provides an exhaustive potential energy landscape of the catalytic Mitsunobu reaction^14^ (Figure 2A). Briefly, catalyst **1a** is protonated by the pronucleophile to give **1b** (**I** → **II**), which dehydrates to give cyclic phosphonium salt **1c** (**II** → **III**)**.** ^55^ The alcohol coordinates **1c**, forming complex **1d** (**III** → **IV**), which rearranges to give the active alkoxyphosphonium carboxylate species **1e** (**IV** → **V**). Finally, **1e** undergoes an Arbuzov-type nucleophilic coupling^56^ to form the inverted ester product and regenerate catalyst **1a** (**V** → **VI**). Two large barriers are apparent: a barrier of 23.3 kcal mol^–1^ for the dehydration of protonated **1b** to form cyclic phosphonium salt **1c** (**II** → **III**), and a barrier of 21.4 kcal mol^–1^ (32.6 kcal mol^–1^ relative to the zero point, **II**) for the nucleophilic coupling of **1e** to give ester product RNu and regenerate catalyst **1a** (**V** → **VI**). From this diagram, as concluded by Houk and co-workers,^41^ it is clear that the final barrier (**V** → **VI**) should be rate-limiting.

By grouping the reaction steps according to barrier heights and molecularity, we constructed a kinetic reaction network to describe the catalytic Mitsunobu reaction using five catalyst states (**1a–e**) and the thermodynamic and kinetic parameters provided by Houk and co-workers^41^ (Figure 2C, see Supporting Information Sections S2 and S3 for details). The uncatalyzed reaction was accounted for in a similar manner. For simplicity, the minor competing Fischer esterification, which can proceed under similar conditions though with retention of stereochemistry, was ignored in our model, in line with Houk and co-worker’s calculations.^41^ We note that Houk’s calculations^41^ included an entropy correction term, thus allowing reasonable approximations of both first and second order rate constants directly based on the calculated barriers (by Eyring analysis).

Denton and co-workers reported critical use of a Dean–Stark apparatus to remove water (molecular sieves, Na_2_SO_4_, and MgSO_4_ were ineffective), citing hydrolytic sensitivity of intermediates **III**–**V**.^14^ Water removal was not considered in the computational analysis, although Houk’s landscape confirms the hydrolytic sensitivity: the backward quenching reaction from dehydrated intermediates to the hydrated catalyst (i.e., **V** → **IV** → **III** → **II**) is strongly thermodynamically (Δ*G*_**V**→**II**_ = −11.2 kcal mol^–1^) and kinetically (Δ*G* ^‡^_**V**→**II**_ = +12.0 kcal mol^–1^ vs Δ*G* ^‡^_**V**→**VI**_ = +21.4 kcal mol^–1^) favored.^41^ Given the evident impact water removal has on the catalytic Mitsunobu reaction, we accounted for this process in our kinetic model with an additional equilibrium between water present in the reaction vessel, and water removed, i.e., caught in the Dean–Stark trap (Figure 2C, bottom right), suspecting it might prove pivotal.

### Kinetic Investigation of the Catalytic Mitsunobu Reaction

Having constructed a kinetic model to describe the catalytic Mitsunobu reaction (Figure 2B), we were able to simulate the expected evolution of species in the reaction. For all simulations, we employed standard reported experimental conditions, with [ROH]_0_ = 0.08 M, [RNu]_0_ = 0.08 M, [**1a**]_0_ = 0.008 M and all other initial concentrations set to 0 M. We initially ignored the removal of water (by setting the rate constants *k*_6_ and *k*_–6_ = 0 s^–1^), and thus simulated the reaction solely according to the parameters obtained from Houk and co-workers’ computational analysis (Figure 3A). Under these conditions, the reaction proceeds very slowly, with only ∼38% yield of product RNu obtained after 24 h, substantially less than the 87–90% yield after 24 h reported experimentally under these conditions in two separate reports,^14,43^ and far from the equilibrium position of >99%. This result confirms the key (neglected) role water removal plays in the reaction kinetics (i.e., water removal is not simply a thermodynamic driving force).

Accordingly, using experimental kinetic data reported by Ling, Zhong and co-workers for RNu formation catalyzed by **1a**,^43^ and keeping the rest of the model fixed, we were able to fit the rate constants for water removal (*k*_6_ (and *k*_–6_), Figure 2B, see Supporting Information, Section S3.2 for details). In order to obtain good agreement between the kinetic model and the data, >99.9% of the water was ultimately removed in the simulation (corresponding to a dryness of 24.6 ppm, see Supporting Information, Section 4.1). Importantly, the rate constant for water removal was found to be relatively small (*k*_6_ = 2.33 × 10^–4^ s^–1^), ∼4 orders of magnitude slower than the next slowest forward (1st order) rate constant (*k*_2_ = 3.79 s^–1^). We validated the fitted parameters of the model by making experimental measurements of the water removal rate (5.3 ± 1.3 × 10^–3^ s^–1^) and extent (by Karl–Fischer titration, 79 ± 28 ppm after 24 h drying) under the precise Dean–Stark setup reported by Denton^14^ (see Supporting Information, Section S1 for details). These values will vary with initial solvent wetness and Dean–Stark efficiency, but are consistent with our model’s predictions. Hence, the close agreement between the simulated rate and experimental data (Figure 3B) indicates that, once the removal of water is accounted for, Houk and co-workers’ computational model allows good prediction of experimental reality. Furthermore, no phosphonium catalyst intermediates (**1c–1e**) are observed in the simulation (<0.005% of total catalyst), in good agreement with experimental observation.^14^ We confirmed our analysis is robust to substantial (1–2 kcal mol^–1^) variations in the barrier heights to account for possible DFT calculation error (see Supporting Information, Section S4).

**Figure 3:**
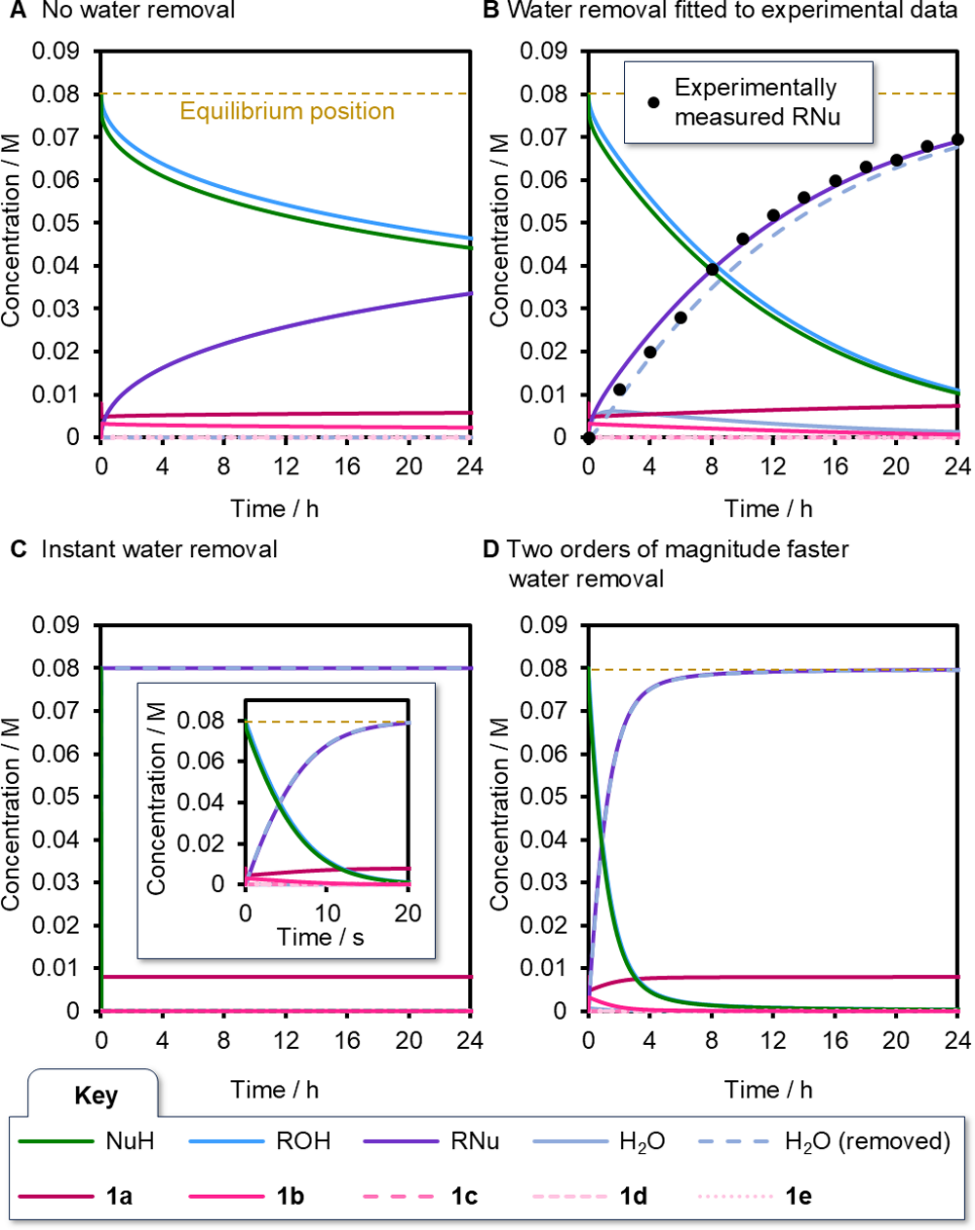
Simulated reaction profiles employing rate constants derived from computational energies for reagents, intermediates and transition states (Figure 2). The yellow dotted line in all panels indicates the equilibrium position of the reaction. (A) Simulated reaction profile with no removal of water from the reaction. (B) Simulated reaction profile generated by fitting the model to experimental data reported by Ling, Zhong and co-workers^43^ to obtain rate constant estimations for water removal (*k*_6_ and *k*_–6_). (C) Simulated reaction profile with effectively instantaneous removal of water from the reaction. (D) Simulated reaction profile with water removal 2 orders of magnitude faster than the experimentally observed rate.

We next systematically varied the rate of water removal in the kinetic simulation. When water is removed effectively instantaneously (*k*_6_ = 10^13^ s^–1^), the reaction reaches equilibrium at >99% conversion to ester within 20 s (Figure 3C). More experimentally plausible water removal rates were also simulated (see Supporting Information, Section S4.2). For example, increasing the rate constant for the removal of water by 2 orders of magnitude (*k*_6_ = 2.33 × 10^–2^ s^–1^) results in a dramatic (approximately 9×) acceleration of the reaction to give >85% yield within 3 h (vs >85% after ∼23 h under the current experimental conditions). Furthermore, we find that, with increased rates of water removal, rapid product formation can be achieved even at significantly lower temperatures (e.g., at 50 °C, >99% yield after just 16 h with 4 orders of magnitude faster water removal, *k*_6_ = 2.33 s^–1^, see Supporting Information, Section S4.3). This result is of great importance since temperature-dependent background reactions currently limit the reaction scope, particularly for activated alcohols.^14^ The profound impact of the rate of water removal on the reaction kinetics raises significant questions as to how to understand the reaction potential energy diagram (Figure 2B). Thus, we sought to develop a model to explain the effect of water removal in order to clarify the disagreement between experiment and theory, and streamline rational design efforts to improve catalyst performance.

### Two-Surface Representation of the Potential Energy Landscape of the Catalytic Mitsunobu Reaction

Having established the significance of water removal in the catalytic Mitsunobu reaction, we next sought to include this vital component in the potential energy landscape representation, since this omission has obscured effective reaction development. Thermodynamically, water removal can be thought of as raising the energy of the hydrated starting materials (states **I** and **II**) relative to the dehydrated products (state ${\mathrm{VI}}^{-H_{2}O}$ ) and intermediates (states ${\mathrm{III}}^{-H_{2}O}$ – $V^{-H_{2}O}$ ), biasing the equilibrium toward product formation (Le Chatelier’s principle).^57,58^ This intuition cannot be readily depicted on a standard potential energy surface and fails to convey kinetic insight; how and why does a change in the equilibrium position as a consequence of Le Chatelier’s principle (thermodynamics) result in a substantial rate enhancement (kinetics)? We now present two visualizations that explain this effect (Figure 4) and so reconcile the misunderstandings resulting from the misleading potential energy diagrams shown in Figure 2.

### Kinetic Barrier Diagrams

First, the removal of water can be included in a kinetic barrier diagram^59,60^ of the reaction (Figure 4A). Briefly, kinetic barrier diagrams incorporate rate constants, reagent concentrations and reaction orders to convey observed relative kinetic barrier heights (i.e., they depict the rate-determining step). We simplified the reaction to remove state **I** and group intermediate states **III**–**V**, thus leaving only the two key barriers for **II** → **III** and **V** → **VI**. Furthermore, we included a kinetic barrier to represent the rate of water removal based on the fitted rate constants (Figure 4A, c.f. Figures 2C and 3B, see Supporting Information, Section S5.6 for details). The first kinetic barrier diagram (Figure 4A­(i)) shows water removal as the highest barrier, unambiguously highlighting the importance of controlling water removal for influencing the reaction. Lowering this barrier (i.e., increasing the rate of water removal/readdition) will clearly result in a rate acceleration, and **V**→**VI** can become rate-limiting (Figure 4A­(ii)). Furthermore, lowering the water removal barrier can also be accompanied by an increased extent of water removal (reaction dryness), effectively lowering all subsequent energy levels (Figure 4A­(iii)). This sort of modification will reduce the rate of **III** → **II** (hydrolysis of the intermediates), thus favoring progression of the reaction to the products. Notably, if the energy levels of states after water removal are dropped sufficiently, then the barrier for $V^{-H_{2}O}$ → ${VI}^{-H_{2}O}$ becomes lower than that for **II** → **III**, representing a change in the rate-determining step of the reaction.

**Figure 4:**
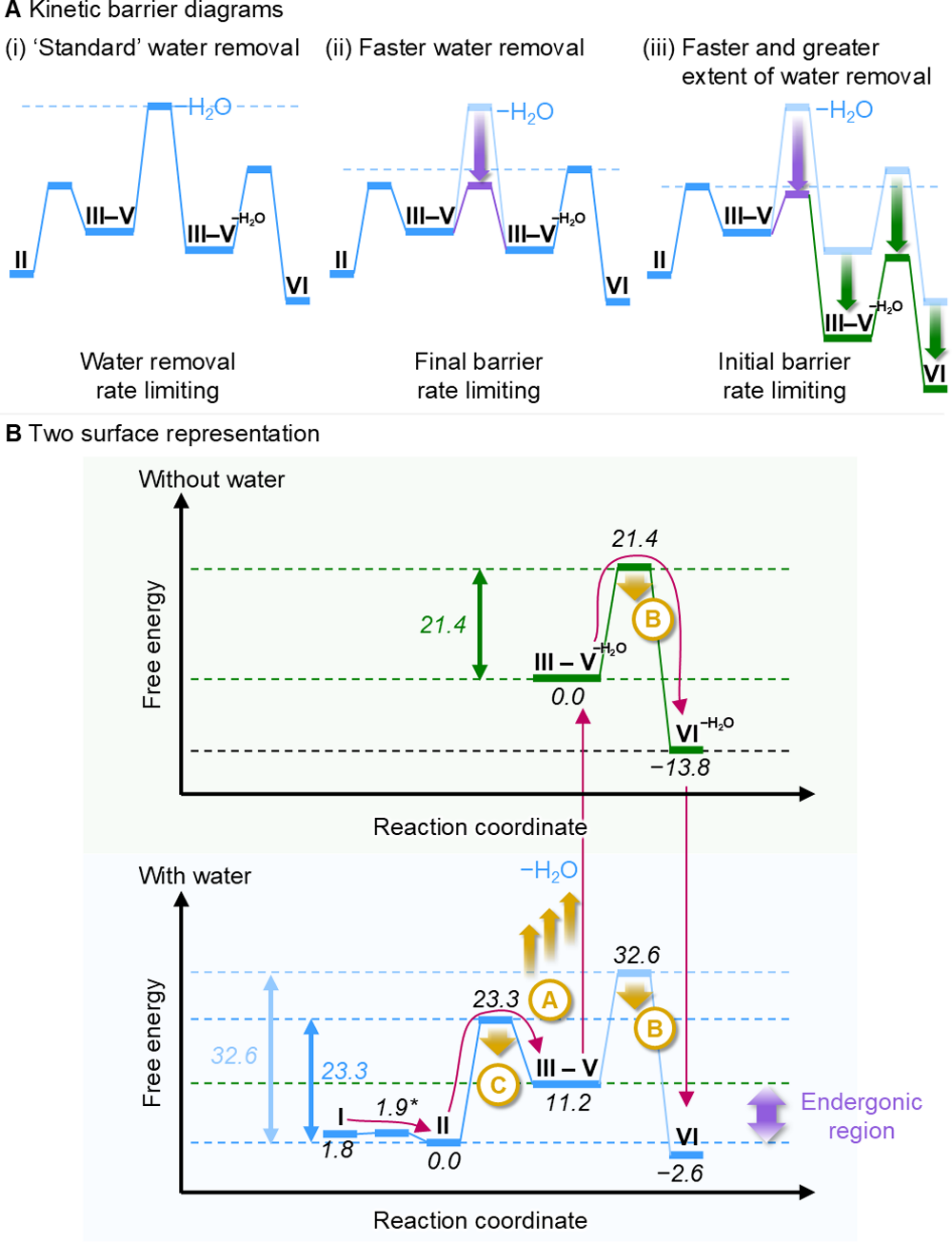
(A) Simplified kinetic barrier diagrams describing the reaction (**I** removed and intermediates **III**–**V** combined). Dotted lines represent the largest kinetic barrier in each case. (i) A kinetic barrier for water removal can be included based on the rate constants determined from fitting experimental data in Figure 3B. This is the highest kinetic barrier in the reaction. (ii) Consequently, lowering the barrier for water removal (increasing the rate of water removal) will accelerate the reaction. (iii) Lowering the barrier to water removal can also be accompanied by an increase in the extent of water removal, which lowers the energy levels for **III**–**V**. If this barrier is sufficiently lowered, then the barrier for **V**–**IV** will be lowered to below the barrier for **II**–**III**. (B) Two-surface representation of the potential energy landscape of the redox-neutral catalytic Mitsunobu reaction (**I** ⇋ **VI**). Energies for all species and transition states are quoted in kcal mol^–1^.^41^ Once generated (step **II** → **III**), water can be physically removed from the reaction from any of states **III**, **IV**, **V**, and **VI**. Removal of water can be thought of as hopping to a new potential energy surface (blue → green), since states **I** and **II** are no longer accessible. Addition of water allows return to the hydrated energy landscape (green → blue). The yellow arrows indicate three potential ways in which the reaction can be accelerated: A, increasing the rate of water removal (blue → green); B, lowering the final nucleophilic coupling barrier (**V** → **VI**); and C, lowering the barrier for catalyst dehydration (**V** → **VI**).

### Two-Surface Representation

An alternative way of visualizing the system is to consider the removal of water as causing the system to hop to a new energy surface where the starting materials are no longer accessible (Figure 4B, blue to green). Thus, after (irreversible) physical water removal, all stationary points have their energies recalibrated to a new “zero-point” and the reaction is necessarily restricted to the new energy surface (Figure 4B, green). Compared to the kinetic barrier diagrams, this two-surface representation loses explicit depiction of the water removal rate, but better depicts systems where water removal is irreversible. Additionally, while the kinetic barrier diagram representation must be recalculated and updated at every time step over the reaction, the two-surface free energy representation offers a simple unchanging overview, depicting the two extremes of “no water removal” and “complete/instant water removal.” Now, one can appraise the relative rate significance of the two first order barriers (dehydration and nucleophilic coupling) by eye. It is immediately apparent that, if the green surface is accessed by fast and irreversible water removal, then the final nucleophilic coupling barrier (Figure 4B, green, $V^{-H_{2}O}$ → ${VI}^{-H_{2}O}$ , Δ*G* ^‡^_**V**→**VI**_ = 21.4 kcal mol^–1^) is no longer rate-determining, and instead chemical dehydration is (Figure 4B, blue, **II** → **III**, Δ*G* ^‡^_**II**→**III**_ = 23.3 kcal mol^–1^, see Supporting Information, Section S4.4 for kinetic simulations). In this case, Houk’s strategy, manipulating the barrier for **V** → **VI**, has a negligible effect on the rate of product formation (see Supporting Information, Section S4.5 for kinetic simulations). This intuition is also visible in the kinetic barrier diagram in Figure 4A­(iii), but the relative importance of the two chemical barriers is obscured by lack of knowledge of the diagram barrier heights at any arbitrary point during the reaction since the barrier heights require full knowledge of reagent concentrations.

In contrast, it is trivial to understand the two-surface representation since it requires only knowledge of the average water concentration (itself a function of water removal rate/efficiency). Then, the relative importance of the two barriers at a given water concentration is equivalent to hypothetical navigation of the two extreme surfaces *by some proportion of the catalyst*. We have plotted the overall reaction rate as a function of water concentration (“chemostated”, see Supporting Information, Section S5), and find that, for catalyst **1a**, the first barrier dominates at water concentrations of ≤0.01 ppm for the current system (see Supporting Information, Section S5). Remarkably, catalyst **2a** need only average 1 ppm water concentration for the first barrier to be rate-limiting (i.e., for the green surface route to contribute more product; see Supporting Information, Section S5). Notably, based on the reported free energy surface for the reaction with catalyst **3a**, only 2 orders of magnitude faster water removal than currently achieved is required to primarily access the green surface route. Finally, this analysis shows that experimentalists should not expect to detect more than ∼ 1% phosphonium salt intermediates (e.g., **1c**) in solvent wetter than ∼0.01 ppm water for the current catalyst/NuH pair at 0.008 M, even in the absence of alcohol.

### Rate-Determining Step of the Catalytic Mitsunobu Reaction

In practice, and as we have established, water removal in current reaction setups is relatively slow (Figure 3B), and so there is a substantial amount of water present in the reaction vessel (up to 5 mM at low reagent conversion, ∼2 equiv relative to the total concentration of dehydrated catalyst species, **1c**, **1d**, and **1e**). This means the reaction *effectively* proceeds to a significant degree on the blue pathway (∼99.999% of the catalyst) where water has not been removed (see Supporting Information, Section S5 for details). Accordingly, artificially lowering the final barrier (**V** → **VI**) in our kinetic model *does* accelerate product formation (see Supporting Information, Section S4.5). Even though the *effective* progression of the reaction by the green potential energy surface is very small (∼0.001% of the catalyst, see Supporting Information, Section S5 for details), this pathway is so much faster that it nonetheless exerts a large influence over the reaction rate (more turnovers) (Figure 4B). Consequently, lowering the final barrier provides a significantly less-pronounced rate enhancement than that predicted by Houk and co-workers. Kinetic simulation of Houk and co-workers’ proposed difluoro catalyst **2a** (Figure 1A, Supporting Information, Section 4.8), which is designed to reduce this final barrier, shows only a ∼90× rate increase over catalyst **1a** under the simulated reaction conditions (as determined by examining the time required to reach 90% product formation), vs the computationally predicted ∼1000× rate increase when water removal is unaccounted for.^41^ This analysis also explains why catalyst **3a** displayed only a modest experimental improvement in rate of reaction and conversion over **1a** (∼13% increase over 20 h).^43^ Our analysis also accounts for the reported partial reaction order in catalyst **1a**,^44^ which typically indicates a competing inactive/quenched catalyst state. Thus, our discussion concerning water removal reconciles the experimental observations with the computational analysis.

### Reaction Design Principles

The corollary of our analysis is clear: the rate of the redox-neutral catalytic Mitsunobu reaction can be increased in three distinct ways as clearly visualized through the two-surface model (Figure 4B, yellow) and as exemplified through the full kinetic models:

First, by increasing the rate of physical water removal from the system (Figure 4A­(ii), Figure 4B, arrow A). This method is always beneficial since water removal *effectively* provides access to the second (green) energy landscape, which has lower barriers than the first (blue) energy landscape (see Supporting Information, Section S4.2).
Second, as proposed by Houk and co-workers, by reducing the final nucleophilic coupling barrier (**V** → **VI**, Figure 4B, arrow B). This method is beneficial when water removal is slow relative to the slowest chemical steps (e.g., as experimentally observed, Figure 3B) since reactions that *effectively* sample only the blue potential energy surface will have their rate increased by reducing the largest barrier (see Supporting Information, Section S4.5).
Third, by reducing the barrier to catalyst dehydration (**II** → **III,** Figure 4B, arrow C). This method is beneficial when water removal is fast relative to the slowest chemical steps since, under conditions where reactions *effectively* sample both the blue and green potential energy surfaces (Figure 4B, pink pathway), this initial barrier to catalyst dehydration becomes the largest barrier, see Supporting Information, Section S4.4). Alternatively, this can be viewed with the aid of kinetic barrier diagrams as the point at which water removal lowers the barrier for $V^{-H_{2}O}$ → ${VI}^{-H_{2}O}$ below the energy of the transitions state for **II** → **III** (Figure 4A­(iii)).

### Experimental Demonstration of Water Removal Effectiveness

The key aspect of our findings is that improving the water removal rate might be a more effective approach to accelerating the reaction than many catalyst modifications.^41,43^ For instance, if the effective concentration of water is maintained at 12.2 ppm under otherwise typical conditions in xylenes, then the reaction using **1a** reaches 50% completion in 1 h instead of 8.3 h (see Supporting Information, Section S5). We sought to experimentally test the water removal hypothesis, and thus performed a model catalytic Mitsunobu reaction (Figure 5A).

We selected toluene as a solvent (bp ∼30 °C lower than xylenes) thus providing a relatively slow reaction, allowing us to demonstrate improvement of industrially relevant processes at reduced temperatures. Employing standard reaction conditions ([ROH]_0_ = 0.08 M, [RNu]_0_ = 0.08 M, [**1a**]_0_ = 0.016 M, toluene, 130 °C, 20 h) and the previously reported Dean–Stark drying approach, we obtained product conversion of 10% (average of 3 repeats) after 20 h (Figure 5B­(i), blue, see Supporting Information, Section S6). Measurements of the concentration of water at the start and end of the reaction by Karl–Fischer titration showed a final water concentration of ∼85 ppm (Figure 5B­(ii)), while fitting the conversion and water concentration data to the kinetic model gave a rate constant of 5.0 × 10^–5^ s^–1^ for water removal under these conditions (Figure 5B­(ii), see Supporting Information, Section S6.1).

Use of an alternative drying approach, where 3 Å molecular sieves were suspended above the refluxing reaction (Figure 3B, green, full details of experimental procedures in Supporting Information, Section S6.1) showed a product conversion of 15% (average of 3 repeats) after 20 h, a remarkable 50% increase in conversion as a result of changing the drying technique. Karl–Fisher titrations to measure the water content confirmed that the overhead desiccant dried the reaction more-extensively (final water concentration ∼12 ppm, Figure 5B­(ii)) and faster (rate constant for water removal = 1.1 × 10^–4^ s^–1^, Figure 5B­(ii)). Kinetic modeling shows that both these factors are required to account for the improvement in product conversion (Supporting Information, Section S6.2). This rate enhancement through the modified drying technique can thus be viewed either as lowering the water removal barrier and subsequent energy levels on a kinetic barrier diagram (Figure 4A­(iii)), or as driving the reaction more to the green energy surface in the two-surface representation (Figure 4B).

**Figure 5:**
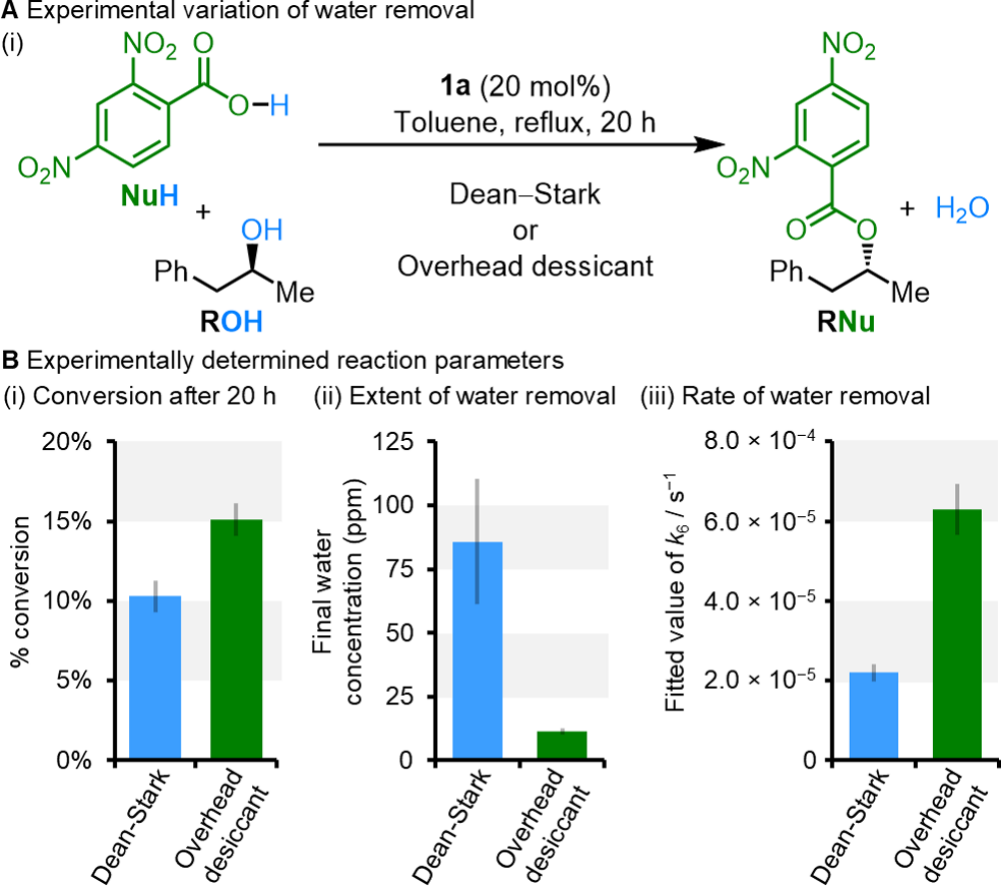
(A) Experimental investigation of the influence of water removal on the catalytic Mitsunobu reaction performed between (*R*/*S*)-1-phenylpropan-2-ol and 2,4-dinitrobenzoic acid with **1a** (20 mol %) in toluene at 111 °C with two drying techniques: Dean–Stark or overhead desiccant (3 Å molecular sieves suspended above the refluxing reaction, see Supporting Information, Section S6.1 for experimental details). (B) Reaction parameters determined for the two drying techniques (error bars given as a single standard deviation based on three repeats, see Supporting Information, Section S6.1 for details). (i) The conversion to product after 20 h. (ii) The final water content of the reaction after 20 h as determined by Karl–Fischer titration. (iii) The rate of water removal as determined from fitting the kinetic model to experimentally determined data (see Supporting Information, Section S6.2, error bars given as a standard 10% error).

### Endergonic Synthesis and Parallels to Ratchet Mechanisms

One intriguing aspect of the two-surface representation of the catalytic Mitsunobu reaction is that it becomes evident that the reaction can be used to drive endergonic reactions (i.e., positive Δ*G* for the equilibrium between **I** ⇋ **VI**, Figures 2 and 4). In principle, removal of a product will drive any equilibrium in this manner through Le Chatelier’s principle, regardless of the energies of the reagents and products. However, for the catalytic redox-neutral Mitsunobu reaction, endergonic processes (Δ*G*_**I**⇋**VI**_ > 0 kcal mol^–1^) can be readily driven on a meaningful time scale since the removal of water perturbs the equilibrium position of the formation of a reactive intermediate (**II** ⇋ **III**). The water removal, depicted as the transition to the green potential energy surface (Figure 4B), prevents hydrolysis of intermediates, and thus formation of product **VI** occurs without (or with reduced) competing hydrolysis. Indeed, kinetic simulations demonstrate that raising the energy of product **VI** from −2.6 to +5 kcal mol^–1^ results in efficient formation of an endergonic product (∼50% conversion after 24 h under standard experimental conditions, see Supporting Information, Section S4.6). Furthermore, as previously discussed for exergonic processes, the rate of the *endergonic* process can be greatly accelerated by increasing the rate of water removal (>96% conversion after 24 h with 2 orders of magnitude faster water removal, see Supporting Information, Section S4.6). In contrast, even with effectively instantaneous water removal, the rate of the *exergonic* uncatalyzed reaction is unchanged (<0.001% conversion after 24 h).

We note that despite the interesting kinetic consideration of this phenomenon, it is essentially a manifestation of Le Chatelier’s principle,^57,58^ and falls short of true endergonic synthesis where energy is transduced through a ratchet mechanism.^51,52,54^ Nonetheless, the parallels to ratcheted endergonic synthesis^61–63^ are notable, and consideration of a detailed kinetic analysis^64^ of the catalytic Mitsunobu reaction serves as a powerful descriptor that can inform the optimization and design of future, related reactions.^65,66^

## Conclusions

In summary, we have performed a rigorous kinetic analysis of Denton’s catalytic Mitsunobu reaction using thermodynamic and kinetic parameters from reported DFT calculations. By constructing a kinetic model that includes a term for the previously ignored water-removal process, we were able to accurately describe experimental data, supported by our own experimental measurements. Through systematic variation of model parameters, most notably the rate of water removal, we were able to make several testable predictions to improve the reaction design. To clarify the kinetic discussion, we have described the reaction using both kinetic barrier diagrams (Figure 4A) and two potential energy surfaces, traversed by the addition/removal of water (Figure 4B), providing an orthogonal layer of control, and demonstrating that the previously reported DFT model alone is insufficient to predict experimental kinetics. Our analysis prompts three areas for improved reaction design: reducing the final nucleophilic coupling barrier, increasing the rate of water removal, and—if water removal can be significantly improved—reducing the barrier to dehydration.

Our findings suggest that, in addition to computationally guided catalyst design,^41,67,68^ increasing the rate of water removal will be crucial to improving the redox-neutral catalytic Mitsunobu reaction and enhancing its industrial utility. The overhead desiccant approach (Figure 5) has allowed us to already realize a 50% rate enhancement of the basic reaction as a consequence of enhanced water removal. New flow technologies,^69^ improved drying systems or azeotropes,^70,71^ membrane reactors capable of drying solvents to <10 ppm water, combined with solid-supported catalysts^50^ could result in processes proceeding at significantly lower temperatures, which will enable scope expansion to more sensitive substrates.

Finally, we note that our findings and the two-surface description of the reaction applies generally to any process where water or gas is evolved in a reaction^65,66^ and removed from the system. Moreover, our two-surface approach indicates a general principle for catalyst/reaction design;^22^ preventing reactive intermediates from reverting to starting materials by rapid removal of a byproduct (e.g., through compartmentalization/flow) results in significant rate enhancements. Thus, we believe that our analysis should provide fundamental and general insights into catalyst design across a wide range of chemistries.

## Supplementary Material



## Notes

Mathematical models, kinetic parameters, simulation graphs, experimental rate constants and graphs, additional experimental procedures (PDF)
