# Supplementary material for: Kinetic Analysis of the Redox-Neutral Catalytic Mitsunobu Reaction: Dehydration, Kinetic Barriers, and Hopping between Potential Energy Surfaces
Source: J Am Chem Soc. 2025 May 13;147(21):18240–8. doi: 10.1021/jacs.5c05404 (PMC12123607; doi:10.1021/jacs.5c05404)
Supplement: Supplementary file 1 [file ja5c05404_si_001.pdf]

Kinetic analysis of the redox-neutral catalytic Mitsunobu reaction: dehydration, kinetic barriers and hopping between potential energy surfaces

Keith G. Andrews*[1] and Stefan Borsley**[1]

[1]Department of Chemistry, Durham University, Lower Mount Joy, South Road, Durham DH1 3LE, United Kingdom

*E-mail: keith.g.andrews@durham.ac.uk

**E-mail: stefan.h.borsley@durham.ac.uk

– Supplementary Information –

Contents

S1. Dean–Stark experimental measurements 3
S1.1 Determination of extent of reaction drying by Karl–Fischer titration 3
S1.2 Experimental determination of rate of water removal 4
S2. Notes on the extraction of rate constants from computational analysis 6
S3. A kinetic model based on a chemical reaction network 8
S3.1 Construction of the kinetic model 8
S3.2 Fitting to experimental data to obtain a rate of water removal 9
S3.3 Limitations of the kinetic model 10
S4. Simulations 11
S4.1 Varying the extent of water removal 11
S4.2 Varying the rate of water removal 12
S4.3 Varying the reaction temperature and water removal rate 14
S4.4 Varying the barrier for II→III 17
S4.5 Varying the barrier for V→VI 19
S4.6 Endergonic product formation 21
S4.7 Simulation of catalyst 2a 22
S5. Simulated profiles at constant water concentration 24
S5.1 A model for constant water concentration 24
S5.2 Simulated reaction profiles at various fixed water concentrations 25
S5.3 Analysis and discussion of fixed water concentration data 26
S5.4 The two-surface model – estimating the proportion of reaction pathways 27
S5.5 Estimation of the water concentration at which II→III becomes rate-limiting 28
S5.6 Kinetic barrier diagrams 29
S6. Experimental demonstration of the effect of water removal rate and extent on the catalytic Mitsunobu reaction rate 30
S6.1 Dean-Stark trap vs overhead desiccant 30
S6.2 Kinetic analysis of experimental data 31
S7. References 32

S1. Dean–Stark experimental measurements

Unless stated otherwise, reagents were obtained from commercial sources and used without purification. Karl-Fischer titrations were made using a Metrohm 831 Karl-Fischer Coulometer using the standard base-buffered methanolic mixture of iodine and sulfur dioxide.

S1.1 Determination of extent of reaction drying by Karl–Fischer titration

According to the scale and set-up reported previously by Denton and co-workers,[S1] Winchester-grade xylenes (25 mL) was added to a 50 mL B14-necked round-bottomed flask containing a 4×10 mm magnetic stirrer bar and the flask connected to a Dean–Stark apparatus and a condenser open to air via a rubber septum with a needle (0.8 mm bore). Distilled water (36 mg, 2.00 mmol) was added, and the reaction stirred at reflux (sand bath at >160 °C) open to atmosphere for 24 h. After this time, the reaction flask was sealed and allowed to cool. Once the reaction vessel had cooled to 25 °C, a known mass of solvent from the reaction flask (0.5–1 mL) was assessed for water content by Karl-Fischer titration. Three repeat measurements were performed per Dean–Stark drying experiment. Three Karl–Fischer titration measurements were also performed on the solvent at the top of the Dean-Stark trap. This whole process was repeated three times to yield the following water content data (Table S1). The initial water concentration of the Winchester grade xylenes (Fisher Scientific) was (154.0, 136.2, 153.5 ppm = 147.9 ± 11.1 ppm), although we also tested an old bottle of xylenes which had substantially higher water content (761.5, 744.8, 781.9 ppm = 780.8 ± 20.4 ppm).

Table S1. Water content as assessed by Karl–Fischer titrations. Three Dean–Stark experiments (1–3) were performed. The initial water content of the xylenes used for these experiments was 147.9 ± 11.1 ppm.

		water (ppm)		*average water ppm and error per experiment*	
experiment	***reading***	***flask***	***trap***	***flask***	***trap***
1	a	64.3	170.4	65.9 ± 5.4	168.5 ± 6.2
	b	62.0	162.1		
	c	71.5	172.9		
2	a	90.1	127.2	85.1 ± 5.0	125.9 ± 6.3
	b	84.2	130.9		
	c	81.1	119.7		
3	a	83.4	119.7	87.4 ± 6.7	119.6 ± 5.3
	b	84.4	124.4		
	c	94.4	114.8		
	**average (ppm)**	79.5	138.0	79.5	138.0
	**error (ppm)**	±28.3	±59.5	±12.9	±29.1

S1.2 Experimental determination of rate of water removal

According to the scale and set-up reported by Denton and co-workers,[S1] Winchester-grade xylenes (25 mL) was added to a 50 mL B14-necked round-bottomed flask containing a 4×10 mm magnetic stirrer bar and the flask connected to a Dean-Stark apparatus and a condenser open to air via a rubber septum with a needle (0.8 mm bore). Added was distilled water (2.000 mL) and the reaction stirred at reflux (sand bath at >160 °C). The reaction was visually inspected for solvent exchange with the Dean-Stark trap, and a timer started when exchange first began. The volume of water collected in the Dean-Stark trap was monitored as a function of time (Table S2) and plotted and fit to estimate a first-order rate constant for water removal (Figure S1).

Table S2. Rate of water removal under Dean–Stark conditions. Initial conditions: H_2O (~2 mL), xylenes (25 mL), reflux. Three Dean–Stark experiments (1–3) were performed.

	Time / s	H_2O in Dean-Stark trap / mL	H_2O remaining / mL
**Repeat 1**	0	0	2.1
	130	0.4	1.7
	250	0.8	1.3
	340	1	1.1
	390	1.2	0.9
	450	1.3	0.8
	550	1.6	0.5
	670	1.7	0.4
	810	2.1	0
**Repeat 2**	0	0	2.05
	45	0.1	1.95
	96	0.4	1.65
	174	0.8	1.25
	293	1.4	0.65
	380	1.7	0.35
	444	1.9	0.15
	530	2	0.05
	618	2.05	0
**Repeat 3**	0	0	2
	124	0.1	1.9
	187	0.2	1.8
	228	0.3	1.7
	310	0.5	1.5
	370	0.7	1.3
	440	0.9	1.1
	505	1	1
	613	1.3	0.7
	700	1.6	0.4
	738	1.7	0.3
	819	1.9	0.1
	905	2	0

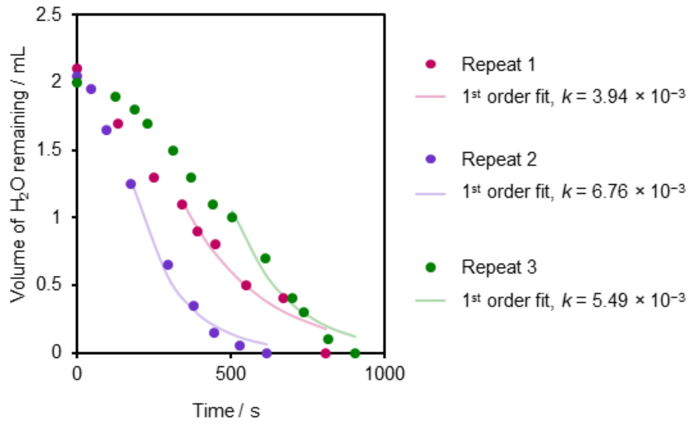

Figure S1. Experimentally measured rate of water removal under Dean−Stark conditions. Experimental data were fitted to a first order rate equation to give an experimentally measured rate constant for water removal of 5.40 ± 1.41 × 10^{-3} s^{-1}.

The kinetic data for the removal of water clearly have substantial error associated. Nonetheless, by discounting the initial few data points, reasonable first order fits can be obtained (Figure S1). The rate profiles look sigmoidal. There are a number of reasons that may explain this. First, the glassware takes time to heat up and equilibrate the temperature throughout the Dean−Stark apparatus. Second, the volume of water employed (~2 mL) phase separates in xylenes (25 mL). It is likely that the evaporation of phase separated and solvated water occurs at different rates, and indeed this may also explain why the rate is slightly faster (~1 order of magnitude) than the rate of water removal employed in the kinetic model to accurately describe the experimental data (see Section S3.2), where the average concentration is much lower, and thus a greater proportion of the water is likely solvated. However, the data nonetheless validate that the rate of water removal in the kinetic model (see Section S3.2) is a reasonable estimate.

S2. Notes on the extraction of rate constants from computational analysis

We employed the potential energy landscape for the redox-neutral catalytic Mitsunobu reaction (catalyzed by **1a**) reported by Houk and co-workers[S2] (Main Text, Figure 2A, reproduced here for convenience as Figure S2). We note that Houk and co-workers described the transformation from **II→III** as a two-step process, however, to facilitate systematic variation of the kinetic model we have coarse grained this process into a single step.

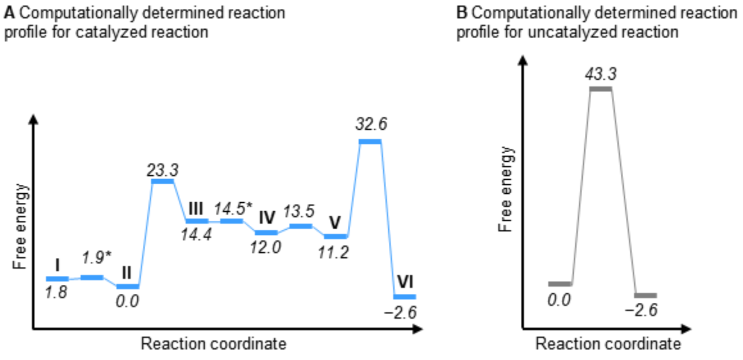

Figure S2. Computationally determined[S2] reaction profiles for (**A**) **1a**-catalyzed and (**B**) uncatalyzed Mitsunobu reaction.

Transition state theory provides a means of converting the computationally determined activation barriers into rate constants (*k*) through the Eyring equation (equation S1)

$$k = \kappa \frac{k_B T}{h} e^{-\frac{\Delta G^{\ddagger}}{RT}} \qquad \textbf{S1}$$

Where R is the ideal gas constant, k_B is the Boltzmann constant, h is Plank's constant and T is the temperature in Kelvin. κ is assumed to be 1. Since the reaction was performed in refluxing xylenes, T was set to 139 °C, the boiling point of xylenes (412.15 K), and the computationally predicted rate constants under the reported experimental reaction conditions are thus presented in Table S3.

Table S3. Computationally determined activation energies ($\Delta\Delta G^{‡}$)[S2] and the corresponding rate constants as determined from transition state theory by application of the Eyring equation at a reaction temperature of at 139 °C (412.15 K, boiling point of xylenes).

Process	Overall reaction	Forward reaction: Calculated energy barrier, $\Delta\Delta G^{‡}$ / kcal mol^{-1}	Forward reaction: Rate constant			Backward reaction: Calculated energy barrier, $\Delta\Delta G^{‡}$ / kcal mol^{-1}	Backward reaction: Rate constant		
**I**→**II**	**1a** + NuH ⇋ **1b**	0.1*	k_1	7.60×10^{12}	s^{-1} M^{-1}	1.9*	k_{-1}	8.44×10^{11}	s^{-1}
**II**→**III**	**1b** ⇋ **1c** + H_2O	23.3	k_2	3.79	s^{-1}	8.9	k_{-2}	1.64×10^{8}	s^{-1} M^{-1}
**III**→**IV**	**1c** + ROH ⇋ **1d**	0.1*	k_3	7.60×10^{12}	s^{-1} M^{-1}	2.5*	k_{-3}	4.06×10^{11}	s^{-1}
**IV**→**V**	**1d** ⇋ **1e**	1.5	k_4	1.38×10^{12}	s^{-1}	2.3	k_{-4}	5.18×10^{11}	s^{-1}
**V**→**VI**	**1e** ⇋ **1a** + RNu	21.4	k_5	38.6	s^{-1}	35.2	k_{-5}	1.86×10^{-6}	s^{-1} M^{-1}
Uncatalyzed	NuH + ROH ⇋ RNu + H_2O	41.5	k_7	8.48×10^{-10}	s^{-1} M^{-1}	45.9	k_{-7}	3.94×10^{-12}	s^{-1} M^{-1}

* No computational barrier was provided for these processes,[S2] which were assumed to have a very low barrier (they are sterically unhindered protic association steps). For the purpose of this analysis we have arbitrarily assigned a 0.1 kcal mol^{-1} barrier for the forward process, which is sufficiently low to ensure that these steps are several orders of magnitude faster than all other steps.

S3. A kinetic model based on a chemical reaction network

S3.1 Construction of the kinetic model

Having extracted rate constants from the computationally determined energy profile of the reaction, we next used COPASI 4.40 (Build 278)[S3] to construct a kinetic model based on the catalytic cycle in Figure 2C (reproduced here for convenience as Figure S3).

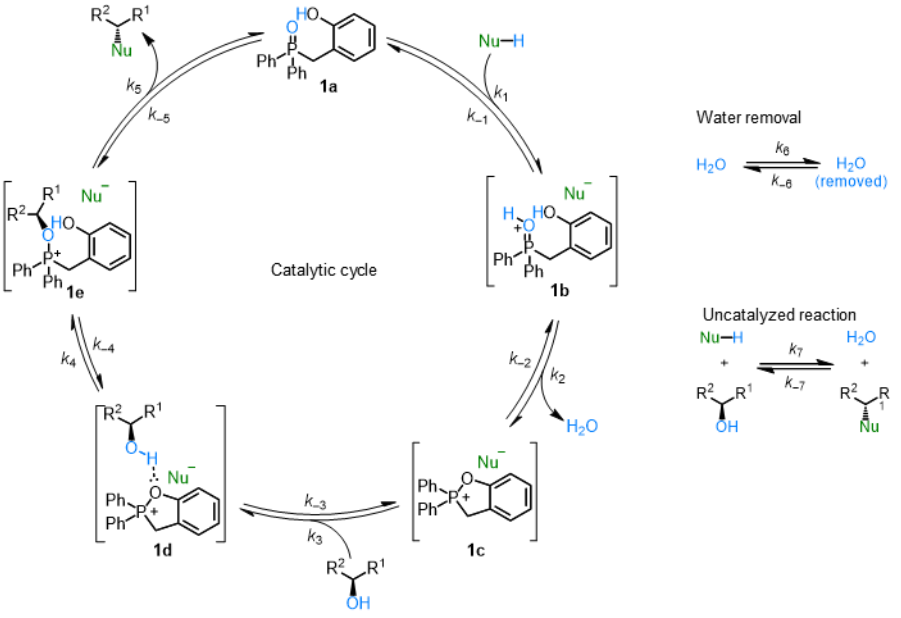

Figure S3. Reproduction of Figure 2c from the main text showing the chemical reaction network and rate constants (k_n/k_{-n}) employed to build the kinetic model outlined by the differential reactions below.

In addition to the simple catalytic cycle and the uncatalyzed background reaction, we included an additional process for the removal of water. For the purposes of the kinetic simulation the water was converted to a new, otherwise unreactive species (H_2O (removed)) through a first order reaction, which is functionally the same as physically removing water from the reaction vessel, which might also be approximated as a first order process.

The differential equations that describe the evolution of species in the reaction network over time are reported below (equations S2–S11).

$$\frac{d[\mathbf{1a}]}{dt} = k_5[\mathbf{1e}] - k_{-5}[\mathbf{1a}][\mathrm{RNu}] - k_1[\mathbf{1a}][\mathrm{NuH}] + k_{-1}[\mathbf{1b}] \quad \text{S2}$$

$$\frac{d[\mathbf{1b}]}{dt} = k_1[\mathbf{1a}][\mathrm{NuH}] - k_{-1}[\mathbf{1b}] - k_2[\mathbf{1b}] + k_{-2}[\mathbf{1c}][\mathrm{H_2O}] \quad \text{S3}$$

$$\frac{d[\mathbf{1c}]}{dt} = k_2[\mathbf{1b}] - k_{-2}[\mathbf{1c}][\mathrm{H_2O}] - k_3[\mathbf{1c}][\mathrm{ROH}] + k_{-3}[\mathbf{1d}] \quad \text{S4}$$

$$\frac{d[\mathbf{1d}]}{dt} = k_3[\mathbf{1c}][\mathrm{ROH}] - k_{-3}[\mathbf{1d}] - k_4[\mathbf{1d}] + k_{-4}[\mathbf{1e}] \quad \text{S5}$$

$$\frac{d[\mathbf{1e}]}{dt} = k_4[\mathbf{1d}] - k_{-4}[\mathbf{1e}] - k_5[\mathbf{1e}] + k_{-5}[\mathbf{1a}][\mathrm{RNu}] \quad \text{S6}$$

$$\frac{d[\mathrm{NuH}]}{dt} = -k_1[\mathbf{1a}][\mathrm{NuH}] + k_{-1}[\mathbf{1b}] - k_7[\mathrm{NuH}][\mathrm{ROH}] + k_{-7}[\mathrm{RNu}][\mathrm{H_2O}] \quad \text{S7}$$

$$\frac{d[\mathrm{ROH}]}{dt} = -k_3[\mathbf{1c}][\mathrm{ROH}] + k_{-3}[\mathbf{1d}] - k_7[\mathrm{NuH}][\mathrm{ROH}] + k_{-7}[\mathrm{RNu}][\mathrm{H_2O}] \quad \text{S8}$$

$$\frac{d[\mathrm{RNu}]}{dt} = k_5[\mathbf{1e}] - k_{-5}[\mathbf{1a}][\mathrm{RNu}] + k_7[\mathrm{NuH}][\mathrm{ROH}] - k_{-7}[\mathrm{RNu}][\mathrm{H_2O}] \quad \text{S9}$$

$$\frac{d[\mathrm{H_2O}]}{dt} = k_2[\mathbf{1b}] - k_{-2}[\mathbf{1c}][\mathrm{H_2O}] + k_7[\mathrm{NuH}][\mathrm{ROH}] - k_{-7}[\mathrm{RNu}][\mathrm{H_2O}] - k_6[\mathrm{H_2O}] + k_{-6}[\mathrm{H_2O\ (removed)}] \quad \text{S10}$$

$$\frac{d[\mathrm{H_2O\ (removed)}]}{dt} = k_6[\mathrm{H_2O}] - k_{-6}[\mathrm{H_2O\ (removed)}] \quad \text{S11}$$

S3.2 Fitting to experimental data to obtain a rate of water removal

The computational analysis provided rate constants for all processes except for the rate of water removal (k_6/k_{-6}). However, the rate constant(s) for this process could be obtained by fitting the model to experimentally determined kinetic data for the evolution of RNu over time.[S4] We performed the fitting in COPASI (kinetic modelling software) through an evolutionary programming method, allowing only the rate constants for the removal/addition of water to be varied, while fixing all other rate constants in the network at the values determined from the computational analysis (see Section S3.1). The rate constants were estimated as $k_6 = 2.33 \times 10^{-4}$ s^{-1} and $k_{-6} = 2.33 \times 10^{-7}$ s^{-1}. The fit indicated a high extent of water removal (99.9% at equilibrium), as determined by the ratio of the forward and reverse rate constants (k_6/k_{-6}). While this reverse rate constant was modelled to ensure thermodynamic consistency, simply setting this rate constant to 0 resulted in no significant worsening of the fit.

S3.3 Limitations of the kinetic model

The ability of the model to faithfully describe experimental data (see main text Figure 3B and Section S3.2) at an experimentally plausible rate of water removal (see Section S1) supports the validity of the model. However, there are a few limitations/approximations that should be noted. Perhaps most notably, the model neglects inclusion of the competing Fischer esterification reaction, which proceeds via a retention mechanism. The high experimental e.e. values show this pathway makes a negligible contribution for the substrates/system modelled here. Likewise, direct hydrolysis of intermediate **V** is not accounted for (though it is likely that this is a slower pathway than going via **IV** and **III**; these transitions are fast enough ($>10^{11}$ s^{-1}) to ensure this approximation does not noticeably impact the model).

We also make several approximations concerning the modelling of the physical Dean–Stark trap. During the data fitting, and typical simulations, we assumed the initial flask/trap solvent reservoir water concentration was zero. This value is experimentally implausible, but the water generated during the experiment exceeds typical initial solvent water concentrations (e.g. as measured in Section S1) by more than an order of magnitude. We demonstrate in Section S5 that, for the current system and the water concentrations observed, the uncertainty in the initial water concentration has a minimal effect on the reaction kinetics over the timescale examined.

This result also justifies our approximation of the Dean–Stark trap in the model. Experimentally, a separation of a bulk water phase in the Dean–Stark trap occurs, affecting the water concentration in the xylenes solvent phase in the trap in a complex manner, and therefore affecting residual water return to the reaction flask. These processes are coarse grained in our model as a single water removal step: we model the trap as a single xylenes phase, with volume equal to the flask. The rate of return of water from trap to flask is sufficiently slow (k_{-6}) that these approximations do not affect the water concentration in the flask significantly, and so the kinetic profile is also unaffected. If the water return process were significantly faster, the model would require a more accurate treatment of phase separation in the trap.

We restate the finding that all our model results are consistent with both the experimental catalysis and experimental water removal data presented within the text.

Finally, while changes to specific rate constants in the model provide a powerful way of exploring the effect of specific processes on the reaction, practically it is generally not possible to affect a single process (e.g. a single reaction barrier) in isolation, hence adjusting the model for different catalysts or reagents should require the energy profile to be re-calculated.

S4. Simulations

Satisfied that the kinetic model provides a good representation of the experimental data, we were able use COPASI[S3] to vary individual rate constants and simulate the expected evolution of species in the reaction over time.

For all simulations the initial conditions were as followed: $[ROH]_0$ = 0.08 M, $[NuH]_0$ = 0.08 M, [**1a**] = 0.008 M, [**1b**] = 0 M, [**1c**] = 0 M, [**1d**] = 0 M, [**1e**] = 0 M, [H_2O] = 0 M, [H_2O (removed)] = 0 M.

S4.1 Varying the extent of water removal

Since we included a reverse rate for the water removal process in the kinetic model, this provides a means to vary the extent of water removal (at equilibrium) from the reaction. We fixed the forward rate constant for water removal (k_6 = 2.33 × 10^{-4} s^{-1}) and varied the reverse rate by ±2 orders of magnitude (Figures S4 and S5).

A

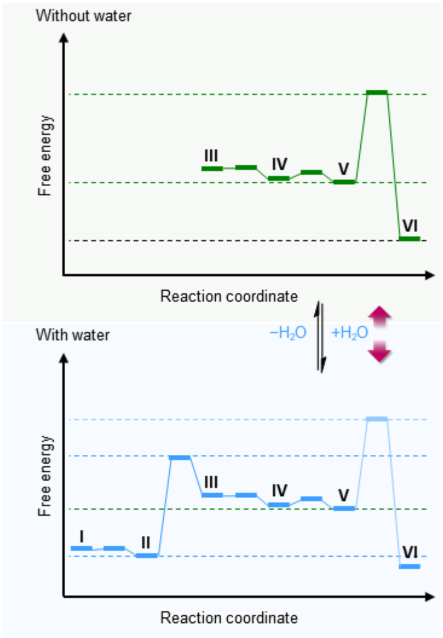

B

Process	Reaction		Forward reaction Rate constant			Backward reaction Rate constant	
I→II	**1a** + NuH ⇋ **1b**	k_1	7.60 × 10^{12}	s^{-1} M^{-1}	k_{-1}	8.44 × 10^{11}	s^{-1}
II→III	**1b** ⇋ **1c** + H_2O	k_2	3.79	s^{-1}	k_{-2}	1.64 × 10^{8}	s^{-1} M^{-1}
III→IV	**1c** + ROH ⇋ **1d**	k_3	7.60 × 10^{12}	s^{-1} M^{-1}	k_{-3}	4.06 × 10^{11}	s^{-1}
IV→V	**1d** ⇋ **1e**	k_4	1.38 × 10^{12}	s^{-1}	k_{-4}	5.18 × 10^{11}	s^{-1}
V→VI	**1e** ⇋ **1a** + RNu	k_5	38.6	s^{-1}	k_{-5}	1.86 × 10^{-6}	s^{-1} M^{-1}
Water removal	H_2O ⇋ H_2O (removed)	k_6	2.33 × 10^{-4}	s^{-1}	k_{-6}	2.33 × 10^{-9}<br>2.33 × 10^{-7}<br>2.33 × 10^{-5}	s^{-1}
Uncatalyzed	NuH + ROH ⇋ RNu + H_2O	k_7	8.48 × 10^{-10}	s^{-1} M^{-1}	k_{-7}	3.94 × 10^{-12}	s^{-1} M^{-1}

Figure S4. (**A**) Illustration of the change in the energy profile of the reaction employed to assess the effect of varying the extent of water removal. (**B**) Rate constants employed in simulations to assess the effect of varying the extent of water removal on the reaction at 139 °C (412.15 K, boiling point of xylenes). Black values indicate original model, while pink values indicate variations to produce reaction profiles in Figure S5.

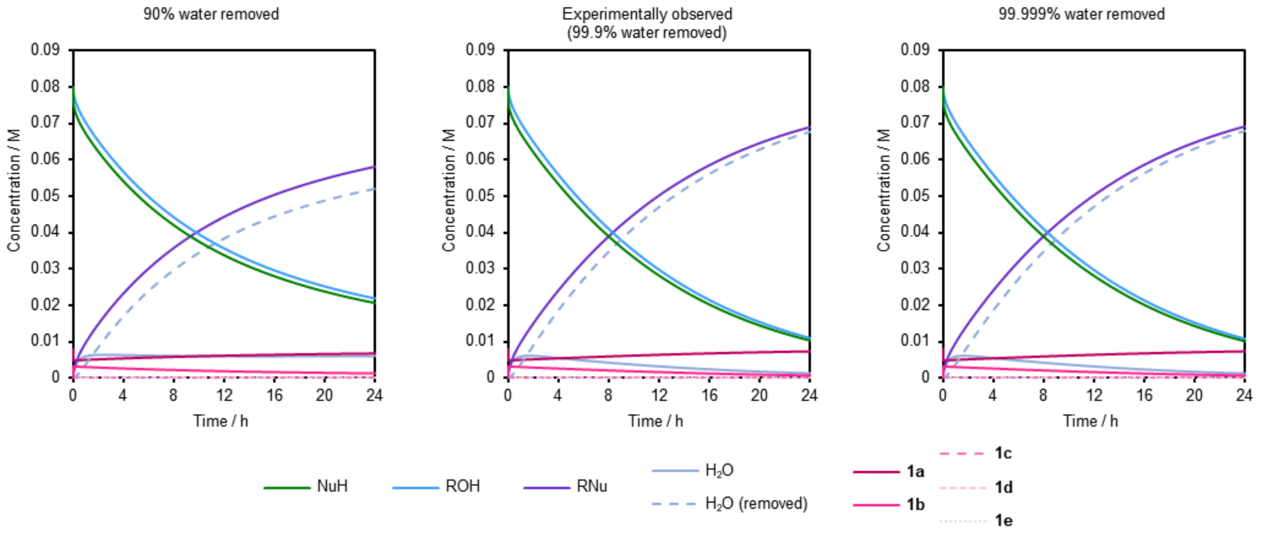

Figure S5. Simulated reaction profiles demonstrating the effect of varying the extent of water removal on the reaction kinetics. Because water removal is slow, drying to 99.999% equilibrium dryness barely affects the kinetic profiles on this timescale.

S4.2 Varying the rate of water removal

The rate of water removal was varied by changing k_6. The corresponding reverse rate constant, k_{-6}, was also changed to maintain a ratio of k_6/k_{-6} = 99.9 (Figures S6 and S7).

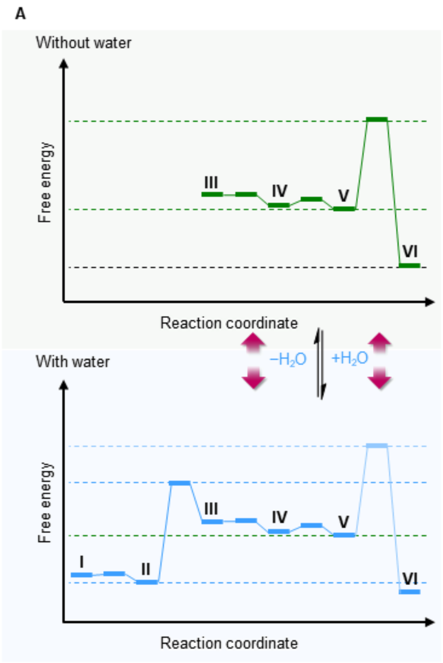

B

Process	Reaction		Forward reaction<br>Rate constant			Backward reaction<br>Rate constant	
**I**→**II**	**1a** + NuH ⇋ **1b**	k_1	7.60 × 10^{12}	s^{-1} M^{-1}	k_{-1}	8.44 × 10^{11}	s^{-1}
**II**→**III**	**1b** ⇋ **1c** + H_2O	k_2	3.79	s^{-1}	k_{-2}	1.64 × 10^{8}	s^{-1} M^{-1}
**III**→**IV**	**1c** + ROH ⇋ **1d**	k_3	7.60 × 10^{12}	s^{-1} M^{-1}	k_{-3}	4.06 × 10^{11}	s^{-1}
**IV**→**V**	**1d** ⇋ **1e**	k_4	1.38 × 10^{12}	s^{-1}	k_{-4}	5.18 × 10^{11}	s^{-1}
**V**→**VI**	**1e** ⇋ **1a** + RNu	k_5	38.6	s^{-1}	k_{-5}	1.86 × 10^{-6}	s^{-1} M^{-1}
Water removal	H_2O ⇋ H_2O (removed)	k_6	2.33 × 10^{-6}<br>2.33 × 10^{-4}<br>2.33 × 10^{-3}<br>2.33 × 10^{-2}<br>2.33 × 10^{-1}<br>23.3	s^{-1}	k_{-6}	2.33 × 10^{-9}<br>2.33 × 10^{-7}<br>2.33 × 10^{-6}<br>2.33 × 10^{-5}<br>2.33 × 10^{-4}<br>2.33 × 10^{-2}	s^{-1}
Uncatalyzed	NuH + ROH ⇋ RNu + H_2O	k_7	8.48 × 10^{-10}	s^{-1} M^{-1}	k_{-7}	3.94 × 10^{-12}	s^{-1} M^{-1}

Figure S6. (**A**) Illustration of the change in the energy profile of the reaction employed to assess the effect of varying the rate of water removal. (**B**) Rate constants employed in simulations to assess the effect of varying the rate of water removal on the reaction at 139 °C (412.15 K, boiling point of xylenes). Black values indicate original model, while pink values indicate variations to produce reaction profiles in Figure S7.

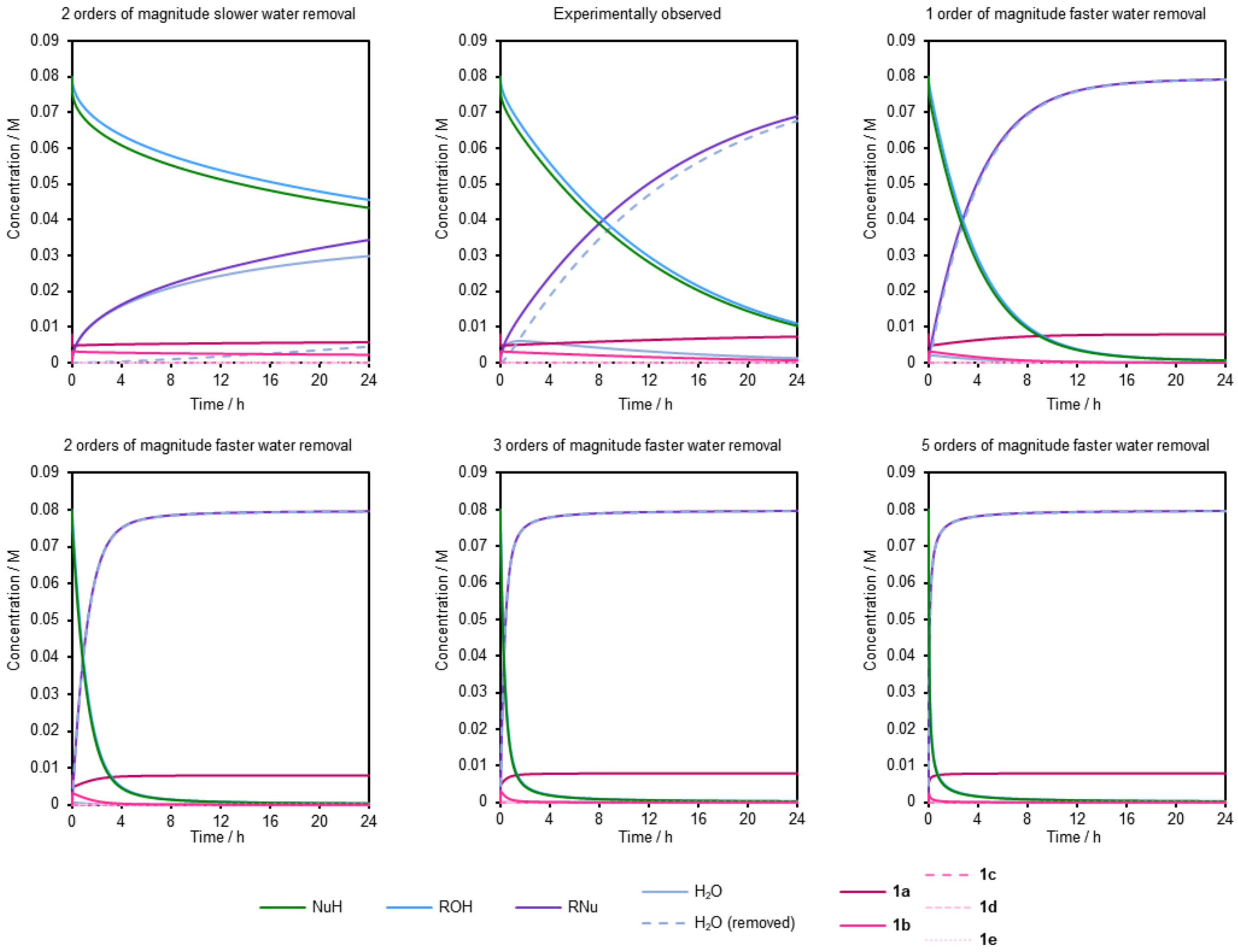

Figure S7. Simulated reaction profiles demonstrating the effect of varying the rate of water removal on the reaction kinetics.

S4.3 Varying the reaction temperature and water removal rate

We re-calculated the rate constants based on the potential energy profile for a range of temperatures and examined what rate of water removal might be necessary to obtain reasonable product conversion at reduced temperatures (50 °C (323.15 K) and 80.74 °C (353.89 K, boiling point of cyclohexane) (Tables S4 and S5).

Table S4. Computationally determined activation energies ($\Delta\Delta G^{‡}$)[S2] and the corresponding rate constants as determined from transition state theory by application of the Eyring equation at a reaction temperature of at 50 °C (323.15 K).

Process	Overall reaction	Forward reaction: Calculated energy barrier, $\Delta\Delta G^{‡}$ / kcal mol^{-1}	Forward reaction: Rate constant		Backward reaction: Calculated energy barrier, $\Delta\Delta G^{‡}$ / kcal mol^{-1}	Backward reaction: Rate constant	
**I**→**II**	**1a** + NuH ⇋ **1b**	0.1*	k_1	5.76×10^{12} s^{-1} M^{-1}	1.9*	k_{-1}	3.49×10^{11} s^{-1}
**II**→**III**	**1b** ⇋ **1c** + H_2O	23.3	k_2	1.18×10^{-3} s^{-1}	8.9	k_{-2}	6.44×10^{6} s^{-1} M^{-1}
**III**→**IV**	**1c** + ROH ⇋ **1d**	0.1*	k_3	5.76×10^{12} s^{-1} M^{-1}	2.5*	k_{-3}	1.37×10^{11} s^{-1}
**IV**→**V**	**1d** ⇋ **1e**	1.5	k_4	6.51×10^{11} s^{-1}	2.3	k_{-4}	1.87×10^{11} s^{-1}
**V**→**VI**	**1e** ⇋ **1a** + RNu	21.4	k_5	2.27×10^{-2} s^{-1}	35.2	k_{-5}	1.05×10^{-11} s^{-1} M^{-1}
Uncatalyzed	NuH + ROH ⇋ RNu + H_2O	41.5	k_7	5.78×10^{-16} s^{-1} M^{-1}	45.9	k_{-7}	6.11×10^{-19} s^{-1} M^{-1}

* No computational barrier was provided for these processes,[S2] which were assumed to have a very low barrier. For the purpose of this analysis we have arbitrarily assigned a 0.1 kcal mol^{-1} barrier for the forward process, which is sufficiently low to ensure that these steps are several orders of magnitude faster than all other steps.

Table S5. Computationally determined activation energies ($\Delta\Delta G^{‡}$)[S2] and the corresponding rate constants as determined from transition state theory by application of the Eyring equation at a reaction temperature of at 80.74 °C (353.89 K, boiling point of cyclohexane).

Process	Overall reaction	Forward reaction: Calculated energy barrier, $\Delta\Delta G^{‡}$ / kcal mol^{-1}	Forward reaction: Rate constant		Backward reaction: Calculated energy barrier, $\Delta\Delta G^{‡}$ / kcal mol^{-1}	Backward reaction: Rate constant	
**I**→**II**	**1a** + NuH ⇋ **1b**	0.1*	k_1	6.40×10^{12} s^{-1} M^{-1}	1.9*	k_{-1}	4.95×10^{11} s^{-1}
**II**→**III**	**1b** ⇋ **1c** + H_2O	23.3	k_2	3.01×10^{-2} s^{-1}	8.9	k_{-2}	3.35×10^{7} s^{-1} M^{-1}
**III**→**IV**	**1c** + ROH ⇋ **1d**	0.1*	k_3	6.40×10^{12} s^{-1} M^{-1}	2.5*	k_{-3}	2.11×10^{11} s^{-1}
**IV**→**V**	**1d** ⇋ **1e**	1.5	k_4	8.74×10^{11} s^{-1}	2.3	k_{-4}	2.80×10^{11} s^{-1}
**V**→**VI**	**1e** ⇋ **1a** + RNu	21.4	k_5	4.49×10^{-1} s^{-1}	35.2	k_{-5}	1.35×10^{-9} s^{-1} M^{-1}
Uncatalyzed	NuH + ROH ⇋ RNu + H_2O	41.5	k_7	1.73×10^{-13} s^{-1} M^{-1}	45.9	k_{-7}	3.33×10^{-16} s^{-1} M^{-1}

* No computational barrier was provided for these processes,[S2] which were assumed to have a very low barrier. For the purpose of this analysis we have arbitrarily assigned a 0.1 kcal mol^{-1} barrier for the forward process, which is sufficiently low to ensure that these steps are several orders of magnitude faster than all other steps.

We next simulated the effect of varying the rate of water removal at 50 °C (323.15 K) and 80.74 °C (353.89 K, boiling point of cyclohexane) by varying k_6. The corresponding reverse rate constant, k_{-6}, was also changed to maintain a ratio of k_6/k_{-6} = 99.9 (Figures S8 and S9).

A 50 °C

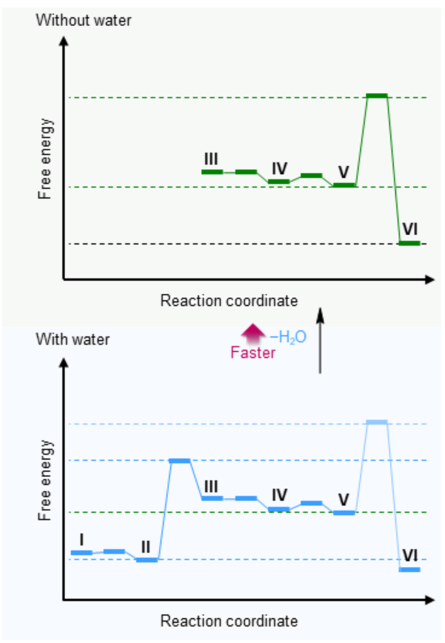

B

Process	Reaction	Forward reaction Rate constant			Backward reaction Rate constant		
I→II	**1a** + NuH ⇋ **1b**	k_1	5.76 × 10^{12}	s^{-1} M^{-1}	k_{-1}	3.49 × 10^{11}	s^{-1}
II→III	**1b** ⇋ **1c** + H_2O	k_2	2.65 × 10^{-2}	s^{-1}	k_{-2}	6.44 × 10^{6}	s^{-1} M^{-1}
III→IV	**1c** + ROH ⇋ **1d**	k_3	5.76 × 10^{12}	s^{-1} M^{-1}	k_{-3}	1.37 × 10^{11}	s^{-1}
IV→V	**1d** ⇋ **1e**	k_4	6.51 × 10^{11}	s^{-1}	k_{-4}	1.87 × 10^{11}	s^{-1}
V→VI	**1e** ⇋ **1a** + RNu	k_5	1.01 × 10^{-3}	s^{-1}	k_{-5}	1.05 × 10^{-11}	s^{-1} M^{-1}
Water removal	H_2O ⇋ H_2O (removed)	k_6	2.33 × 10^{-4}<br>2.33 × 10^{-2}<br>2.33<br>233	s^{-1}	k_{-6}	0	s^{-1}
Uncatalyzed	NuH + ROH ⇋ RNu + H_2O	k_7	5.78 × 10^{-16}	s^{-1} M^{-1}	k_{-7}	6.11 × 10^{-19}	s^{-1} M^{-1}

C

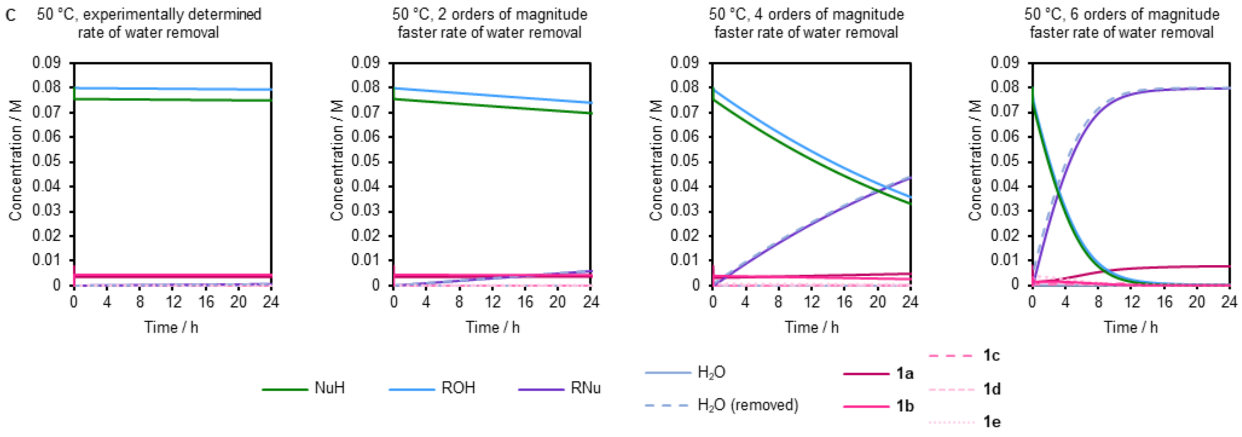

Figure S8. Varying the rate of water removal (k_6) at 50 °C. (**A**) Illustration of the change in the energy profile of the reaction. (**B**) Rate constants employed in simulations. Black values indicate base model, while pink values indicate variations to water removal rate. (**C**) Simulated reaction profiles.

A 80.74 °C (boiling point of cyclohexane)

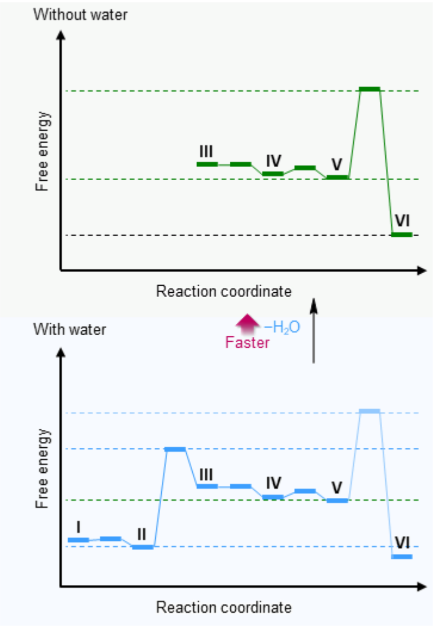

B

Process	Reaction	Forward reaction Rate constant			Backward reaction Rate constant		
I→II	**1a** + NuH ⇋ **1b**	k_1	6.40 × 10^{12}	s^{-1} M^{-1}	k_{-1}	4.95 × 10^{11}	s^{-1}
II→III	**1b** ⇋ **1c** + H_2O	k_2	5.17 × 10^{-1}	s^{-1}	k_{-2}	2.35 × 10^{7}	s^{-1} M^{-1}
III→IV	**1c** + ROH ⇋ **1d**	k_3	6.40 × 10^{12}	s^{-1} M^{-1}	k_{-3}	2.11 × 10^{11}	s^{-1}
IV→V	**1d** ⇋ **1e**	k_4	8.74 × 10^{11}	s^{-1}	k_{-4}	2.80 × 10^{11}	s^{-1}
V→VI	**1e** ⇋ **1a** + RNu	k_5	2.61 × 10^{-2}	s^{-1}	k_{-5}	1.35 × 10^{-9}	s^{-1} M^{-1}
Water removal	H_2O ⇋ H_2O (removed)	k_6	2.33 × 10^{-4}<br>2.33 × 10^{-2}<br>2.33	s^{-1}	k_{-6}	0	s^{-1}
Uncatalyzed	NuH + ROH ⇋ RNu + H_2O	k_7	1.73 × 10^{-13}	s^{-1} M^{-1}	k_{-7}	3.33 × 10^{-16}	s^{-1} M^{-1}

C

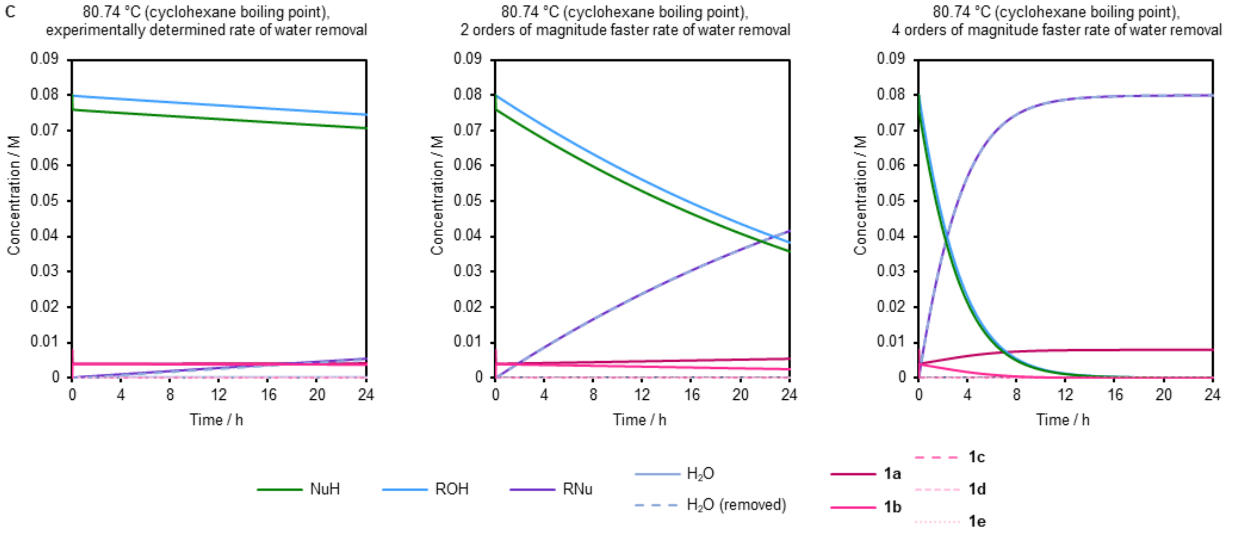

Figure S9. Varying the rate of water removal (k_6) at 80.74 °C (boiling point of cyclohexane). (**A**) Illustration of the change in the energy profile of the reaction. (**B**) Rate constants employed in simulations. Black values indicate base model, while pink values indicate variations to water removal rate. (**C**) Simulated reaction profiles.

S4.4 Varying the barrier for II→III

To simulate the effect of varying the barrier for **II→III**, this barrier was varied by ±2 kcal mol^{-1} while the rest of the potential energy landscape is unchanged. This results in a corresponding change in the rate constants for k_2 and k_{-2} (Figure S10). The resultant rate profiles show no significant change in the kinetic profile of the reaction (Figure S11), in line with Houk and co-workers' assessment.[S2]

A

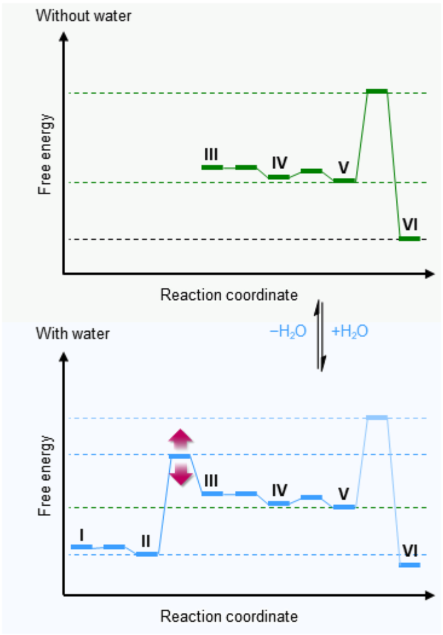

B

Process	Reaction	Forward reaction Rate constant			Backward reaction Rate constant		
I→II	**1a** + NuH ⇋ **1b**	k_1	7.60×10^{12}	s^{-1} M^{-1}	k_{-1}	8.44×10^{11}	s^{-1}
II→III	**1b** ⇋ **1c** + H_2O	k_2	43.6<br>3.79<br>0.33	s^{-1}	k_{-2}	1.88×10^{9}<br>1.64×10^{8}<br>1.43×10^{7}	s^{-1} M^{-1}
III→IV	**1c** + ROH ⇋ **1d**	k_3	7.60×10^{12}	s^{-1} M^{-1}	k_{-3}	4.06×10^{11}	s^{-1}
IV→V	**1d** ⇋ **1e**	k_4	1.38×10^{12}	s^{-1}	k_{-4}	5.18×10^{11}	s^{-1}
V→VI	**1e** ⇋ **1a** + RNu	k_5	38.6	s^{-1}	k_{-5}	1.86×10^{-6}	s^{-1} M^{-1}
Water removal	H_2O ⇋ H_2O (removed)	k_6	2.33×10^{-4}	s^{-1}	k_{-6}	2.33×10^{-7}	s^{-1}
Uncatalyzed	NuH + ROH ⇋ RNu + H_2O	k_7	8.48×10^{-10}	s^{-1} M^{-1}	k_{-7}	3.94×10^{-12}	s^{-1} M^{-1}

Figure S10. (**A**) Illustration of the change in the energy profile of the reaction employed to assess the effect of varying the barrier for **II→III**. (**B**) Rate constants employed in simulations to assess the effect of varying the barrier for **II→III** on the reaction at 139 °C (412.15 K, boiling point of xylenes). Black values indicate original model, while pink values indicate variations to produce reaction profiles in Figure S11.

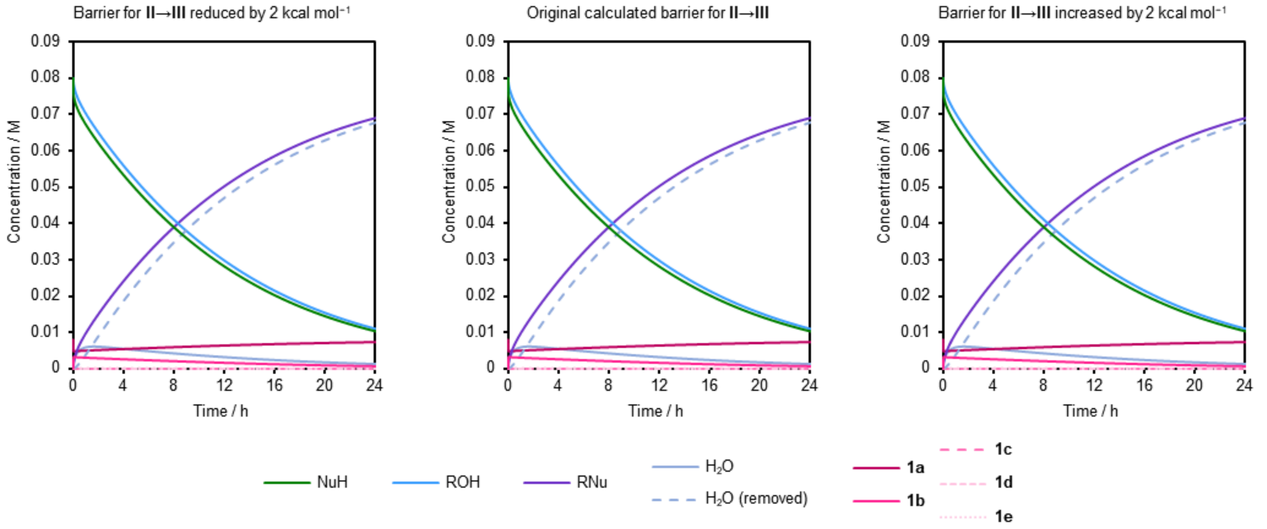

Figure S11. Simulated reaction profiles demonstrating the effect of varying the barrier for **II→III** on the reaction kinetics.

We also simulated similar variation of the barrier for **II**→**III** by ±2 kcal mol^{-1} under conditions where water removal is assumed to be very fast relative to other processes ($k_6 = 10^{13}$ s^{-1}, $k^{-6} = 0$ s^{-1}). Under these conditions, varying the barrier for **II**→**III** has a significant effect on the reaction kinetics (Figure S12). We note that such a water removal rate is unrealistic, though even at slower, more realistic rates this barrier can start to matter (see Section S5).

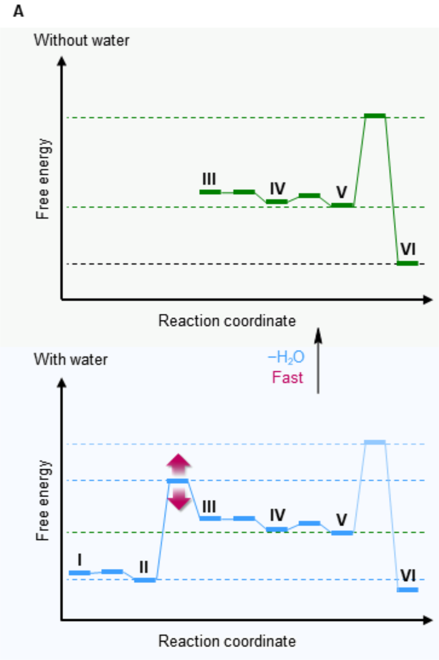

B

Process	Reaction		Forward reaction Rate constant			Backward reaction Rate constant	
I→II	**1a** + NuH ⇋ **1b**	k_1	7.60×10^{12}	s^{-1} M^{-1}	k_{-1}	8.44×10^{11}	s^{-1}
			43.6			1.88×10^{9}	
II→III	**1b** ⇋ **1c** + H_2O	k_2	3.79	s^{-1}	k_{-2}	1.64×10^{8}	s^{-1} M^{-1}
			0.33			1.43×10^{7}	
III→IV	**1c** + ROH ⇋ **1d**	k_3	7.60×10^{12}	s^{-1} M^{-1}	k_{-3}	4.06×10^{11}	s^{-1}
IV→V	**1d** ⇋ **1e**	k_4	1.38×10^{12}	s^{-1}	k_{-4}	5.18×10^{11}	s^{-1}
V→VI	**1e** ⇋ **1a** + RNu	k_5	38.6	s^{-1}	k_{-5}	1.86×10^{-6}	s^{-1} M^{-1}
Water removal	H_2O → H_2O (removed)	k_6	10^{13}	s^{-1}	k_{-6}	0	s^{-1}
Uncatalyzed	NuH + ROH ⇋ RNu + H_2O	k_7	8.48×10^{-10}	s^{-1} M^{-1}	k_{-7}	3.94×10^{-12}	s^{-1} M^{-1}

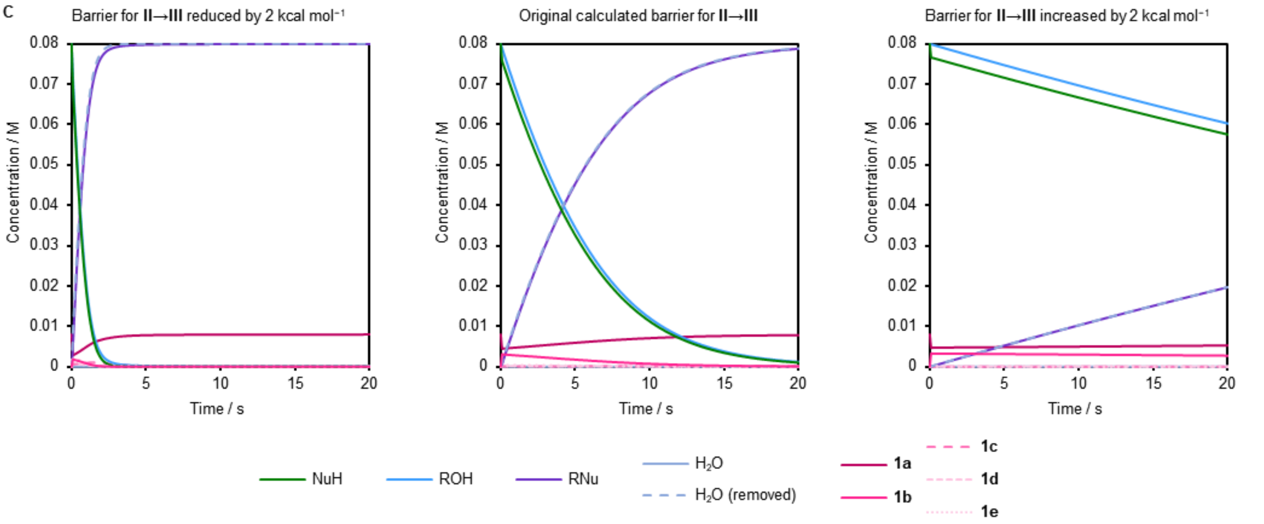

Figure S12. Varying the barrier for **II**→**III** under conditions where water removal is fast relative to other processes ($k_6 = 10^{13}$ s^{-1}, $k_{-6} = 0$ s^{-1}). (**A**) Illustration of the change in the energy profile of the reaction employed to assess the effect of varying the barrier for **II**→**III**. (**B**) Rate constants employed in simulations at 139 °C (412.15 K, boiling point of xylenes). Black values indicate original model, while pink values indicate variations. (**C**) Simulated reaction profiles.

S4.5 Varying the barrier for V→VI

To simulate the effect of varying the barrier for **V→VI**, this barrier was varied by ±2 kcal mol^{-1} while the rest of the potential energy landscape is unchanged. This results in a corresponding change in the rate constants for k_5 and k_{-5} (Figure S13). The resultant rate profiles show a significant change in the kinetic profile of the reaction (Figure S14), in line with Houk and co-workers' assessment.[S2]

A

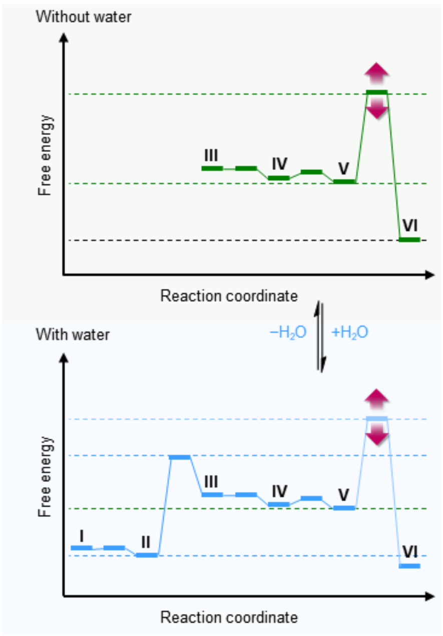

B

Process	Reaction		Forward reaction Rate constant			Backward reaction Rate constant	
I→II	**1a** + NuH ⇋ **1b**	k_1	7.60 × 10^{12}	s^{-1} M^{-1}	k_{-1}	8.44 × 10^{11}	s^{-1}
II→III	**1b** ⇋ **1c** + H_2O	k_2	3.79	s^{-1}	k_{-2}	1.64 × 10^{8}	s^{-1} M^{-1}
III→IV	**1c** + ROH ⇋ **1d**	k_3	7.60 × 10^{12}	s^{-1} M^{-1}	k_{-3}	4.06 × 10^{11}	s^{-1}
IV→V	**1d** ⇋ **1e**	k_4	1.38 × 10^{12}	s^{-1}	k_{-4}	5.18 × 10^{11}	s^{-1}
V→VI	**1e** ⇋ **1a** + RNu	k_5	444<br>38.6<br>3.36	s^{-1}	k_{-5}	2.13 × 10^{-5}<br>1.86 × 10^{-6}<br>1.62 × 10^{-7}	s^{-1} M^{-1}
Water removal	H_2O ⇋ H_2O (removed)	k_6	2.33 × 10^{-4}	s^{-1}	k_{-6}	2.33 × 10^{-7}	s^{-1}
Uncatalyzed	NuH + ROH ⇋ RNu + H_2O	k_7	8.48 × 10^{-10}	s^{-1} M^{-1}	k_{-7}	3.94 × 10^{-12}	s^{-1} M^{-1}

Figure S13. (**A**) Illustration of the change in the energy profile of the reaction employed to assess the effect of varying the barrier for **V→VI**. (**B**) Rate constants employed in simulations to assess the effect of varying the barrier for **V→VI** on the reaction at 139 °C (412.15 K, boiling point of xylenes). Black values indicate original model, while pink values indicate variations to produce reaction profiles in Figure S14.

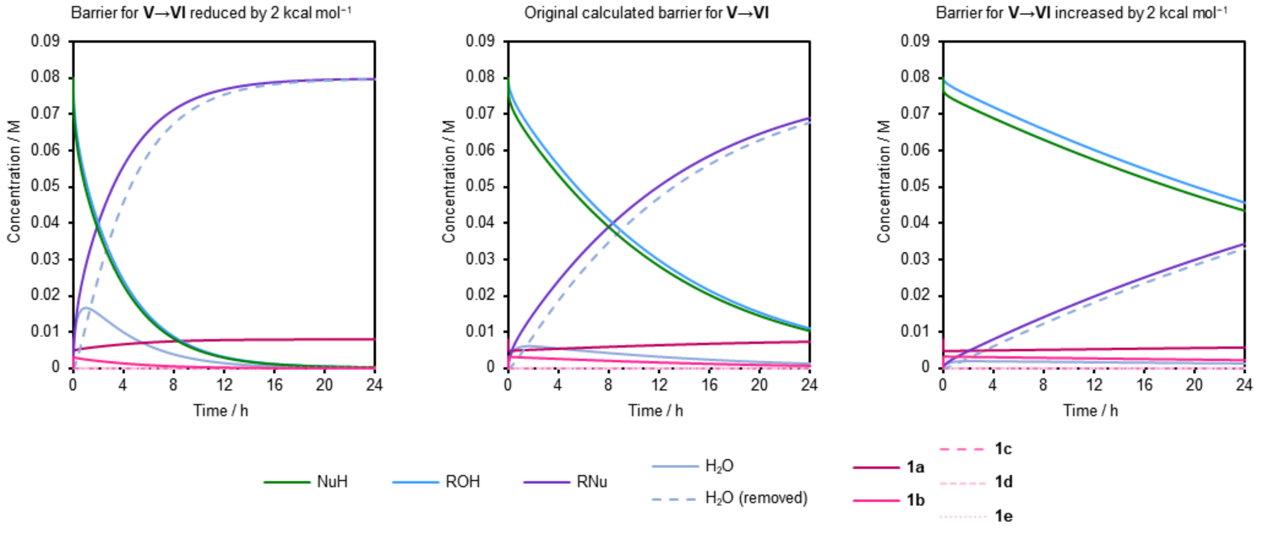

Figure S14. Simulated reaction profiles demonstrating the effect of varying the barrier for **V→VI** on the reaction kinetics.

We also simulated similar variation of the barrier for **V→VI** by ±2 kcal mol^{-1} under conditions where water removal is assumed to be very fast relative to other processes ($k_6 = 10^{13}$ s^{-1}, $k^{-6} = 0$ s^{-1}). Under these conditions, reducing the barrier for **V→VI** has no effect on the reaction kinetics (Figure S15, left). We note that increasing the barrier by 2 kcal mol^{-1} slows the kinetics slightly since this revised barrier is now very similar to the rate limiting barrier for **II→III** (Figure 15C, right).

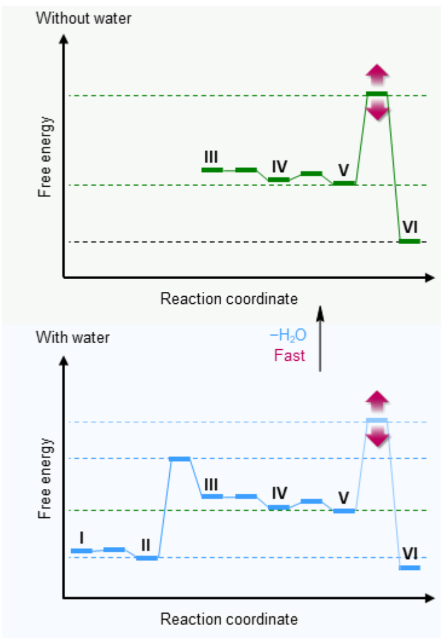

B

Process	Reaction		Forward reaction Rate constant			Backward reaction Rate constant	
I→II	**1a** + NuH ⇋ **1b**	k_1	7.60 × 10^{12}	s^{-1} M^{-1}	k_{-1}	8.44 × 10^{11}	s^{-1}
II→III	**1b** ⇋ **1c** + H_2O	k_2	3.79	s^{-1}	k_{-2}	1.64 × 10^{8}	s^{-1} M^{-1}
III→IV	**1c** + ROH ⇋ **1d**	k_3	7.60 × 10^{12}	s^{-1} M^{-1}	k_{-3}	4.06 × 10^{11}	s^{-1}
IV→V	**1d** ⇋ **1e**	k_4	1.38 × 10^{12}	s^{-1}	k_{-4}	5.18 × 10^{11}	s^{-1}
			444			2.13 × 10^{-5}	
V→VI	**1e** ⇋ **1a** + RNu	k_5	38.6	s^{-1}	k_{-5}	1.86 × 10^{-6}	s^{-1} M^{-1}
			3.36			1.62 × 10^{-7}	
Water removal	H_2O → H_2O (removed)	k_6	10^{13}	s^{-1}	k_{-6}	0	s^{-1}
Uncatalyzed	NuH + ROH ⇋ RNu + H_2O	k_7	8.48 × 10^{-10}	s^{-1} M^{-1}	k_{-7}	3.94 × 10^{-12}	s^{-1} M^{-1}

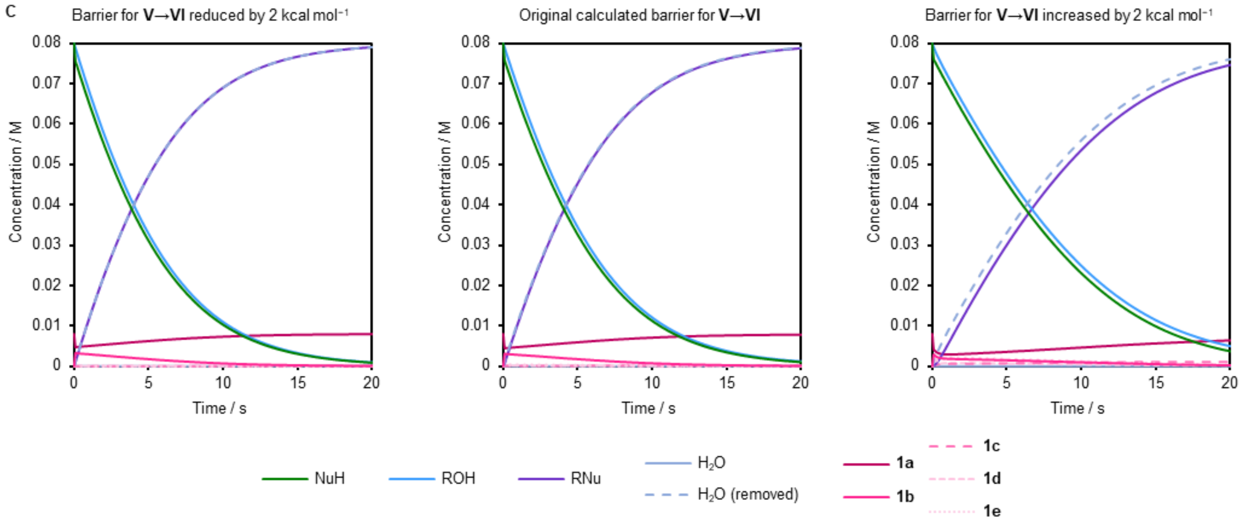

Figure S15. Varying the barrier for **V→VI** under conditions where water removal is fast relative to other processes ($k_6 = 10^{13}$ s^{-1}, $k_{-6} = 0$ s^{-1}). (**A**) Illustration of the change in the energy profile of the reaction employed to assess the effect of varying the barrier for **V→VI**. (**B**) Rate constants employed in simulations at 139 °C (412.15 K, boiling point of xylenes).. (**C**) Simulated reaction profiles.

S4.6 Endergonic product formation

We simulated the effect of increasing the energy of the products from −2.6 to +5 kcal mol^{-1} relative to **II**. The remainder of the potential energy landscape is unchanged. We further explored the effect of varying the rate of water removal on this endergonic reaction.

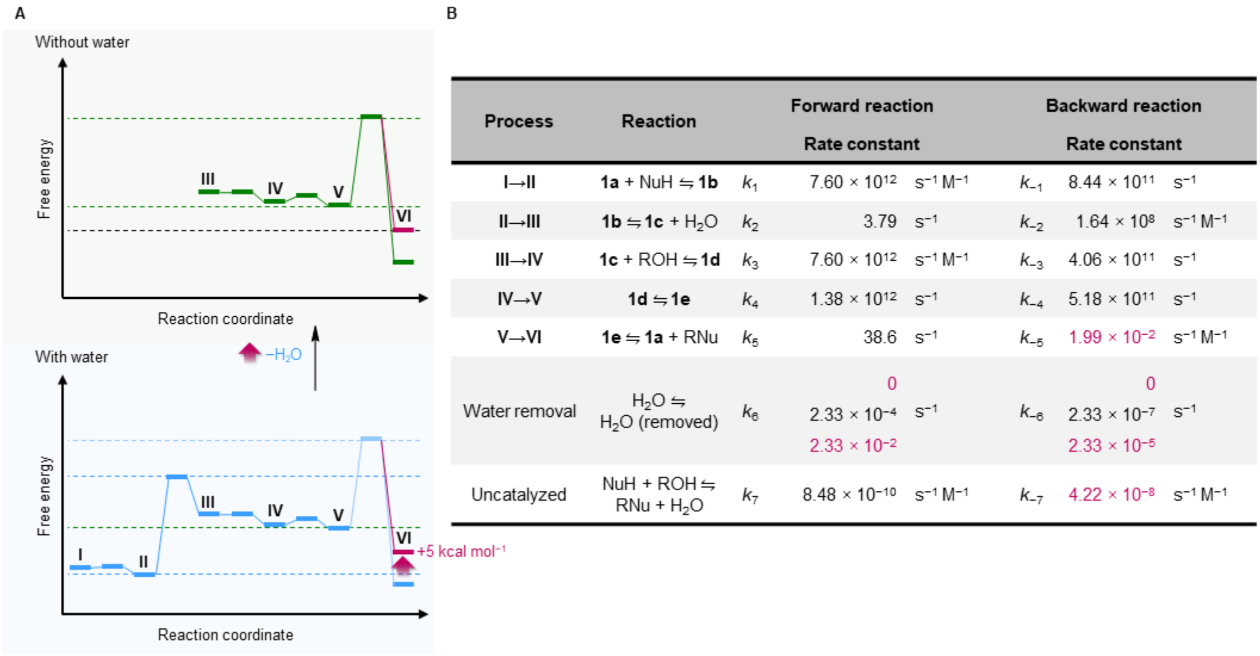

B

Process	Reaction		Forward reaction Rate constant			Backward reaction Rate constant	
I→II	**1a** + NuH ⇋ **1b**	k_1	7.60×10^{12}	$s^{-1} M^{-1}$	k_{-1}	8.44×10^{11}	s^{-1}
II→III	**1b** ⇋ **1c** + H_2O	k_2	3.79	s^{-1}	k_{-2}	1.64×10^{8}	$s^{-1} M^{-1}$
III→IV	**1c** + ROH ⇋ **1d**	k_3	7.60×10^{12}	$s^{-1} M^{-1}$	k_{-3}	4.06×10^{11}	s^{-1}
IV→V	**1d** ⇋ **1e**	k_4	1.38×10^{12}	s^{-1}	k_{-4}	5.18×10^{11}	s^{-1}
V→VI	**1e** ⇋ **1a** + RNu	k_5	38.6	s^{-1}	k_{-5}	1.99×10^{-2}	$s^{-1} M^{-1}$
Water removal	H_2O ⇋ H_2O (removed)	k_6	0<br>2.33×10^{-4}<br>2.33×10^{-2}	s^{-1}	k_{-6}	0<br>2.33×10^{-7}<br>2.33×10^{-5}	s^{-1}
Uncatalyzed	NuH + ROH ⇋ RNu + H_2O	k_7	8.48×10^{-10}	$s^{-1} M^{-1}$	k_{-7}	4.22×10^{-8}	$s^{-1} M^{-1}$

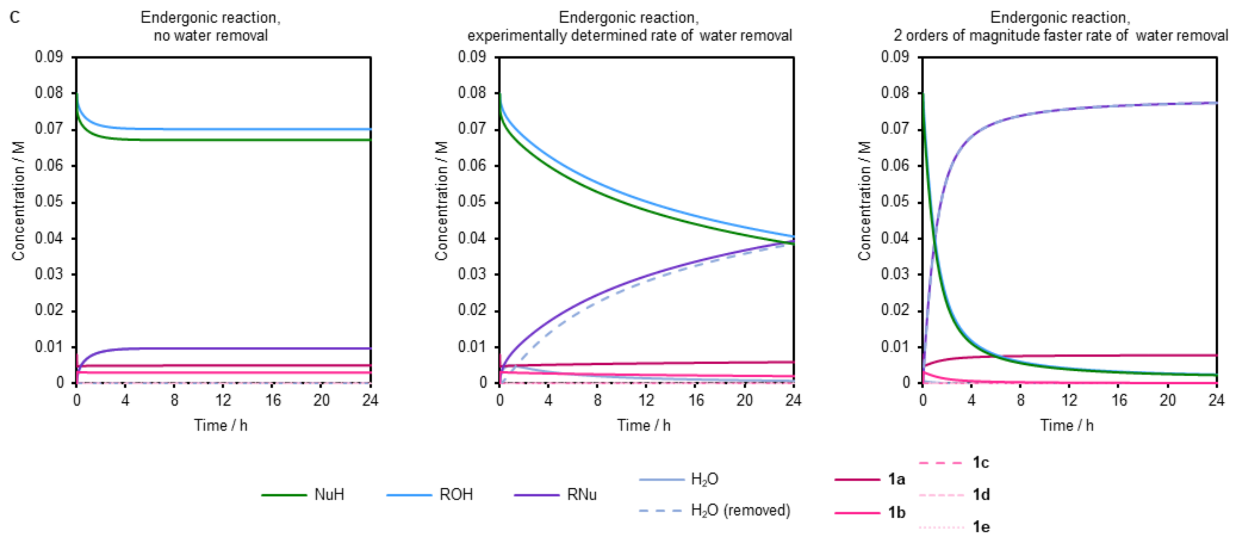

Figure S16. Endergonic product formation. The value of VI was changed from −2.6 to +5 kcal mol^{-1} relative to **II**, which resulted in an increase in the rates of k_{-5} and k_{-7}. Varying the rate of water removal (k_6/k_{-6}). (**A**) Illustration of the change in the energy profile of the reaction employed to assess the effect of varying the rate of water removal on this endergonic reaction. (**B**) Rate constants employed in simulations at 139 °C (412.15 K, boiling point of xylenes). Black values indicate original model, while pink values indicate variations. (**C**) Simulated reaction profiles.

S4.7 Simulation of catalyst 2a

Using the computational values provided by Houk and co-workers[S2] we modelled the reactivity of catalyst **2a** (Table S6 and Figure S17) at 139 °C (412.15 K, boiling point of xylenes). We note that detailed calculations were only provided for barriers **II→III** and **V→VI**,[S2] and so all other barriers were assumed to be the same as for **1a**. While this is likely a poor assumption, it is likely that these other barriers remain small relative to **II→III** and **V→VI**, and thus they will not influence the reaction kinetics.

Table S6. Computationally determined activation energies ($\Delta\Delta G^{‡}$)[S2] and the corresponding rate constants for catalyst **2a** as determined from transition state theory by application of the Eyring equation at a reaction temperature of at 139 °C (412.15 K).

Process	Overall reaction	Forward reaction: Calculated energy barrier, $\Delta\Delta G^{‡}$ / kcal mol^{-1}	Forward reaction: Rate constant			Backward reaction: Calculated energy barrier, $\Delta\Delta G^{‡}$ / kcal mol^{-1}	Backward reaction: Rate constant		
**I→II**	**2a** + NuH ⇋ **1b**	0.1*	k_1	7.60×10^{12}	s^{-1} M^{-1}	1.9*	k_{-1}	8.44×10^{11}	s^{-1}
**II→III**	**2b** ⇋ **2c** + H_2O	22.0	k_2	18.5	s^{-1}	7.6	k_{-2}	8.02×10^{8}	s^{-1} M^{-1}
**III→IV**	**2c** + ROH ⇋ **2d**	0.1*	k_3	7.60×10^{12}	s^{-1} M^{-1}	2.5*	k_{-3}	4.06×10^{11}	s^{-1}
**IV→V**	**2d** ⇋ **2e**	1.5†	k_4	1.38×10^{12}	s^{-1}	2.3†	k_{-4}	5.18×10^{11}	s^{-1}
**V→VI**	**2e** ⇋ **2a** + RNu	16.4	k_5	1.73×10^{4}	s^{-1}	30.2	k_{-5}	8.32×10^{-4}	s^{-1} M^{-1}
Uncatalyzed	NuH + ROH ⇋ RNu + H_2O	41.5	k_7	8.48×10^{-10}	s^{-1} M^{-1}	45.9	k_{-7}	3.94×10^{-12}	s^{-1} M^{-1}

* No computational barrier was provided for these processes, which were assumed to have a very low barrier. For the purpose of this analysis we have arbitrarily assigned a 0.1 kcal mol^{-1} barrier for the forward process, which is sufficiently low to ensure that these steps are several orders of magnitude faster than all other steps. † These values were not provided, and so are treated as per catalyst **1a**.

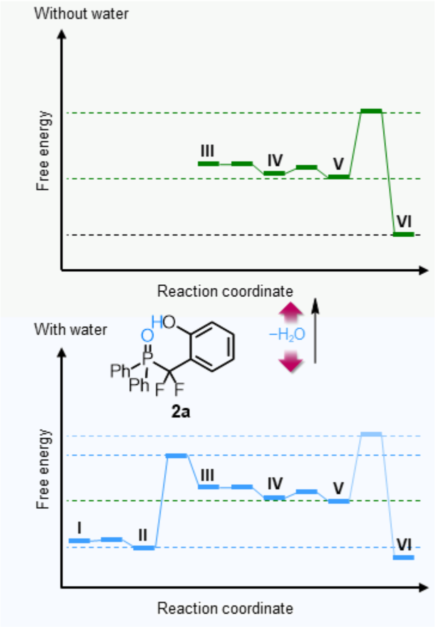

Process	Reaction		Forward reaction Rate constant			Backward reaction Rate constant	
I→II	**2a** + NuH ⇋ **2b**	k_1	7.60×10^{12}	$s^{-1}\,M^{-1}$	k_{-1}	8.44×10^{11}	s^{-1}
II→III	**2b** ⇋ **2c** + H_2O	k_2	18.5	s^{-1}	k_{-2}	8.02×10^{8}	$s^{-1}\,M^{-1}$
III→IV	**2c** + ROH ⇋ **2d**	k_3	7.60×10^{12}	$s^{-1}\,M^{-1}$	k_{-3}	4.06×10^{11}	s^{-1}
IV→V	**2d** ⇋ **2e**	k_4	1.38×10^{12}	s^{-1}	k_{-4}	5.18×10^{11}	s^{-1}
V→VI	**2e** ⇋ **2a** + RNu	k_5	1.73×10^{4}	s^{-1}	k_{-5}	8.32×10^{-4}	$s^{-1}\,M^{-1}$
Water removal	H_2O ⇋ H_2O (removed)	k_6	0<br>2.33×10^{-4}<br>2.33×10^{-2}	s^{-1}	k_{-6}	0<br>2.33×10^{-7}<br>2.33×10^{-5}	s^{-1}
Uncatalyzed	NuH + ROH ⇋ RNu + H_2O	k_7	8.48×10^{-10}	$s^{-1}\,M^{-1}$	k_{-7}	3.94×10^{-12}	$s^{-1}\,M^{-1}$

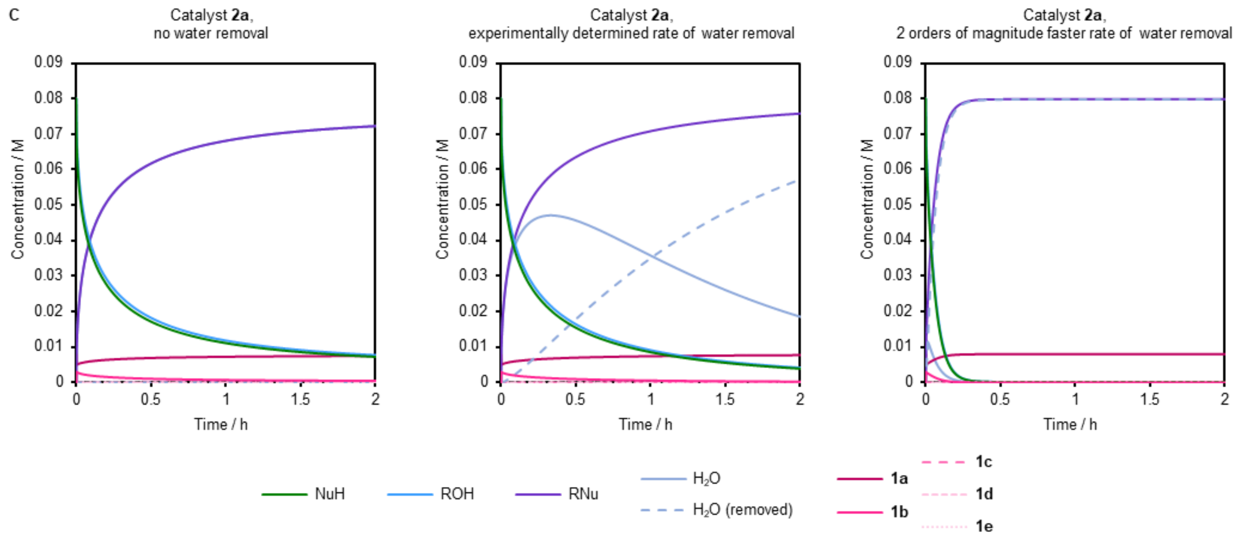

Figure S17. Reaction profiles were simulated for catalyst **2a** based on computationally-determined rate constants (Table S6). The rate of water removal (k_6/k_{-6}) was varied. (**A**) Illustration of the change in the energy profile of the reaction employed to assess the effect of varying barriers **II→III** and **V→VI**. (**B**) Rate constants employed in simulations at 139 °C (412.15 K, boiling point of xylenes). Black values indicate original model, while pink values indicate variations. (**C**) Simulated reaction profiles.

S5. Simulated profiles at constant water concentration

S5.1 A model for constant water concentration

The reaction model was modified to provide reaction profiles at fixed water concentrations (Table S7). In practice, we modified the original model (equations **S2–S11**) in the following way:

1. The water removal constants, k_6 and k_{-6} were set to 0 s^{-1}.
2. The initial internal water concentration was set to non-zero values (see Table S8).
3. Process **II⇋III** was split into two irreversible steps for the forward and backward rates, so that the fate of the water could be independently controlled. The forward process, **II→III**, was set to produce H_2O (removed) directly, while the reverse process, **III→II** was made to produce a molecule of water. Consequently, the water concentration was kept constant in the model.
4. To avoid a drift in the concentration of water, the very slow uncatalyzed reaction was ignored (k_7 and k_{-7} were set to 0). This did not affect the overall profiles noticeably.

Table S7. Model for simulating Denton's redox-neutral Mitsunobu reaction[S1] with catalyst **1a** at constant water concentration, with rate constants as determined from transition state theory by application of the Eyring equation at a reaction temperature of at 25 °C (298.15 K) using Houk and co-workers' computationally determined activation energies ($\Delta\Delta G^{‡}$).[S2]

Process	Overall reaction	Forward reaction Rate constant		Backward reaction Rate constant	
**I→II**	**1a** + NuH ⇋ **1b**	k_1	5.25×10^{12} s^{-1} M^{-1}	k_{-1}	2.52×10^{11} s^{-1}
**II→III**	**1b** → **1c** + H_2O (removed)	k_2	5.18×10^{-5} s^{-1}		
**III→II**	**1c** + H_2O → **1b** + H_2O	k_{-2}	1.86×10^{6} s^{-1}		
**III→IV**	**1c** + ROH ⇋ **1d**	k_3	5.25×10^{12} s^{-1} M^{-1}	k_{-3}	9.14×10^{10} s^{-1}
**IV→V**	**1d** ⇋ **1e**	k_4	4.94×10^{11} s^{-1}	k_{-4}	1.28×10^{11} s^{-1}
**V→VI**	**1e** ⇋ **1a** + RNu	k_5	1.28×10^{-3} s^{-1}	k_{-5}	9.81×10^{-14} s^{-1} M^{-1}
Water removal	H_2O ⇋ H_2O (removed)	k_6	0 s^{-1}	k_{-6}	0 s^{-1}
Uncatalyzed	NuH + ROH ⇋ RNu + H_2O	k_7	0 s^{-1} M^{-1}	k_{-7}	0 s^{-1} M^{-1}

In the overall reaction column, "→" denotes an irreversible process.

S5.2 Simulated reaction profiles at various fixed water concentrations

The water concentration was varied between 0–20 mol% and reaction profiles were simulated according to the model in S5.1. The following representative plots were generated (Figure S18):

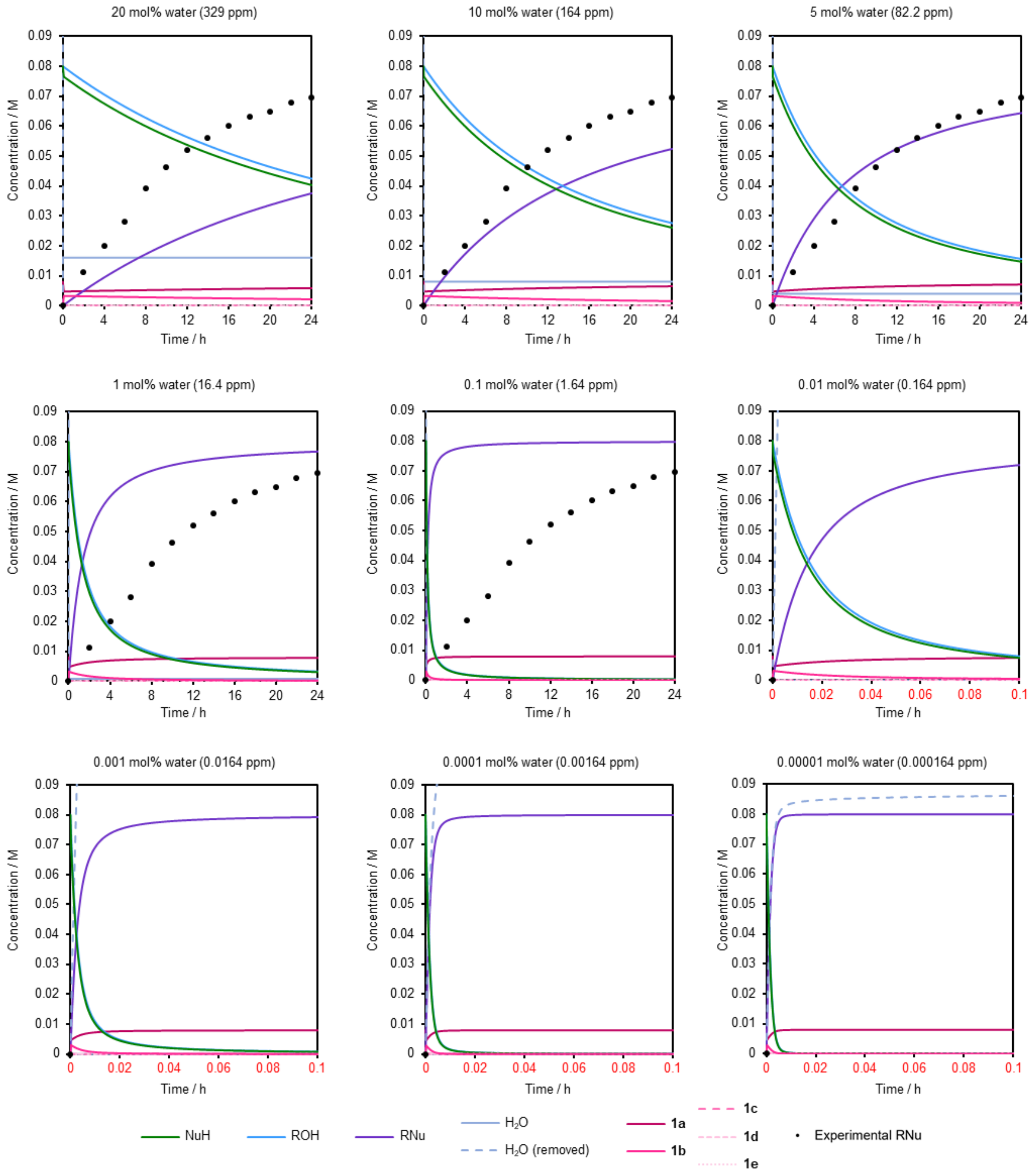

Figure S18. Simulated reaction profiles demonstrating the effect of varying the fixed concentration of water on the reaction kinetics according to the kinetic model presented in Section S5.1. Black dots indicate the experimentally determined reaction profile.[S4] The X-axis in the final four graphs is reduced from 24 h to just 0.1 h, highlighted in red for clarity.

The lowest experimentally measured water content in this study (79.5 ppm, see Section S1.1) corresponds most closely to the simulation modelling a constant water concentration of 82.2 ppm (5 mol% water, Figure S16, top right). We note that the shape of the simulated curves (under the assumption of constant water concentration) does not match the experimental data well, highlighting the fact that *the rate of water removal* is the pertinent term under non-instantaneous (experimental) Dean-Stark conditions. These data may be more relevant under different water-removal approaches.

S5.3 Analysis and discussion of fixed water concentration data

The data shown in the plots in **S5.2** were analyzed. The time taken to reach 50% conversion ($t_{(50\%)}$ s) was extracted from each set of data (interpolating linearly between relevant timesteps (1 s for fast reactions; 90 s for slow reactions) where necessary). The rate of ester (NuR) formation at that time point (d[NuR]/$dt_{(50\%)}$) was found by dividing the product molarity at that point (0.08 M × 0.5 = 0.04 M) by ($t_{(50\%)}$ / s).

Table S8. Simulated kinetic data under different constant water concentrations. $t_{(50\%)}$ is the time in seconds taken to reach 50% conversion to ester (NuR). d[NuR]/$dt_{(50\%)}$ is the rate of product formation (× 10^6 / M s^{-1}) at $t_{(50\%)}$. The selection of this conversion point is effectively arbitrary, and has no effect on the conclusions drawn in section **S5.4**, and minor effects on the conclusions in section **S5.5**, as discussed therein.

[H_2O] / M	H_2O (ppm)	H_2O mol%	$t_{(50\%)}$ / s	d[NuR]/$dt_{(50\%)}$ × 10^6 / M s^{-1}
0	0.00000	0	4.257	9396
8.0×10^{-10}	0.00002	0.000001	4.262	9385
8.0×10^{-9}	0.0002	0.00001	4.307	9288
8.0×10^{-8}	0.0016	0.0001	4.739	8440
8.0×10^{-7}	0.0164	0.001	9.074	4408
8.0×10^{-6}	0.164	0.01	52.56	761.0
8.0×10^{-5}	1.64	0.1	489.3	81.74
8.0×10^{-4}	16.4	1	4836	8.271
1.6×10^{-3}	32.9	2	9668	4.137
2.4×10^{-3}	49.3	3	14501	2.759
3.2×10^{-3}	65.8	4	19333	2.069
4.0×10^{-3}	82.2	5	24167	1.655
4.8×10^{-3}	98.6	6	29000	1.379
5.6×10^{-3}	115	7	33834	1.182
6.4×10^{-3}	132	8	38669	1.034
7.2×10^{-3}	148	9	43503	0.919
8.0×10^{-3}	164	10	48338	0.827
1.6×10^{-2}	329	20	96710	0.414

S5.4 The two-surface model – estimating the proportion of reaction pathways

The ratio k_2[**1b**] / k_{-2}[**1c+1d+1e**][H_2O] is constant over the course of the reaction at a fixed constant concentration of water. This ratio therefore determines the overall reaction rate within this model, and decays as a power function with respect to increasing water concentration. A log–log plot of the ratio k_2[**1b**] / k_{-2}[**1c+1d+1e**][H_2O] against water concentration therefore gives a straight line. This allows an estimation to be made of the extent to which the reaction proceeds on the green or blue potential energy surfaces within the two-surface model (Main Text, Figure 4). When the forward rate is greater than the backward rate (k_2[**1b**] > k_{-2}[**1c+1d+1e**][H_2O]) then the reaction can be viewed as proceeding mostly on the green surface (the forward reaction of the activated catalysts **1c**,**1d**,**1e** is faster than their quenching to reform **1b**, which is equivalent to the green potential energy surface where there can be no backwards reaction.) In contrast, when the backwards rate is faster, as is the case experimentally (k_2[**1b**] < k_{-2}[**1c+1d+1e**][H_2O]) the reaction occurs mostly on the blue potential energy surface. We restate here, for clarity, that the two-surface model (Main Text, Figure 4) is a useful fiction, which is only strictly valid under the assumption of instant and complete water removal, but nonetheless provides valuable insight.

We can estimate the greatest probable experimental ratio (i.e. the extent to which the reaction proceeds on the green surface) by approximating the water concentration as the final dryness measured experimentally (79.5 ppm, see Table S1). This point is labelled in Figure S19, indicating the likely highest green/blue ratio achieved is 6×10^{-5}. Thus, for catalyst **1**, to access mostly the green surface requires the water concentration to be 0.00484 ppm, equivalent to improving the water dryness by 4–5 orders of magnitude. Notably, as the barrier to nucleophilic coupling (**V**→**VI**) is reduced, the required dryness for the surface crossover point will decrease. Performing the same analysis for catalyst **2**, brings the crossover point to approximately 0.5 ppm water, which is likely within the regime of experimentally accessible dryness.

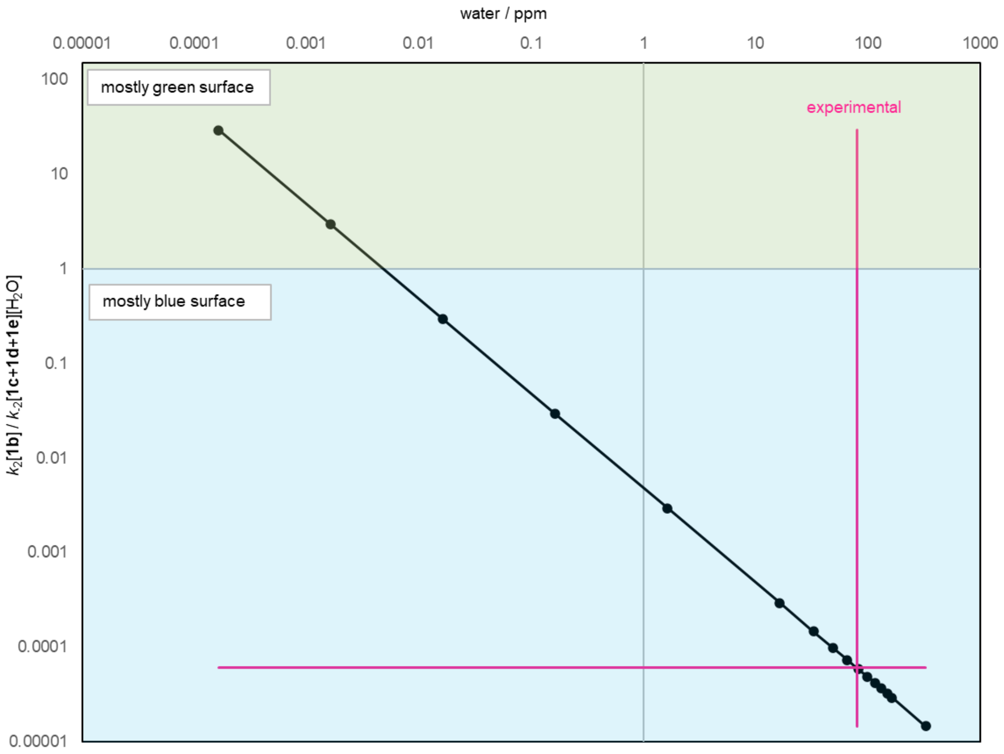

Figure S19. Plot of the ratio of the rate of hydrolysis of dehydrated catalyst species **III**–**V**→**II** vs the rate of nucleophilic coupling **V**→**VI** (k_2[**1b**] / k_{-2}[**1c+1d+1e**][H_2O]) against a constant water concentration in ppm for a reaction at 0.08 M ROH and NuH and 10 mol% catalyst **1a** in 25 mL xylenes.

S5.5 Estimation of the water concentration at which II→III becomes rate-limiting

Using the data in Table S8 (*d*[NuR]/*dt*$_{(50\%)}$ as a function of water concentration), the concentration of water at which the dehydration barrier (**II**→**III**) becomes rate-limiting can be identified. If the data are plotted on a log–log scale, two linear segments are found with a curve between them characteristic of a change in rate determining step. The dehydration barrier (**II**→**III**) is rate-limiting when the water concentration is too low to dominate equation **S4**, which is restated here:

$$\frac{d[\mathbf{1c}]}{dt} = k_2[\mathbf{1b}] - k_{-2}[\mathbf{1c}][\mathrm{H_2O}] - k_3[\mathbf{1c}][\mathrm{ROH}] + k_{-3}[\mathbf{1d}] \qquad \textbf{S4}$$

The overall rate of product formation, *d*[NuR]/*dt*, therefore approaches an asymptote at the maximal rate at vanishing water concentration (equivalent to the instant water removal and zero-water concentration model). This analysis is valid under the assumption of instant water removal and a steady-state concentration of water.

In contrast, at larger [H_2O], the k_{-2} term dominates, and the forward rate decreases. The result is a linear fit on a log-log plot with the power law fit solved at 50% conversion as:

$$\frac{d[\mathrm{NuR}]}{dt_{50\%}} = 1.3 \times 10^{-5}\ [\mathrm{H_2O}]\ ppm^{-0.991} \qquad \textbf{S12}$$

In other words, the reaction order is negative (−0.991) in water.

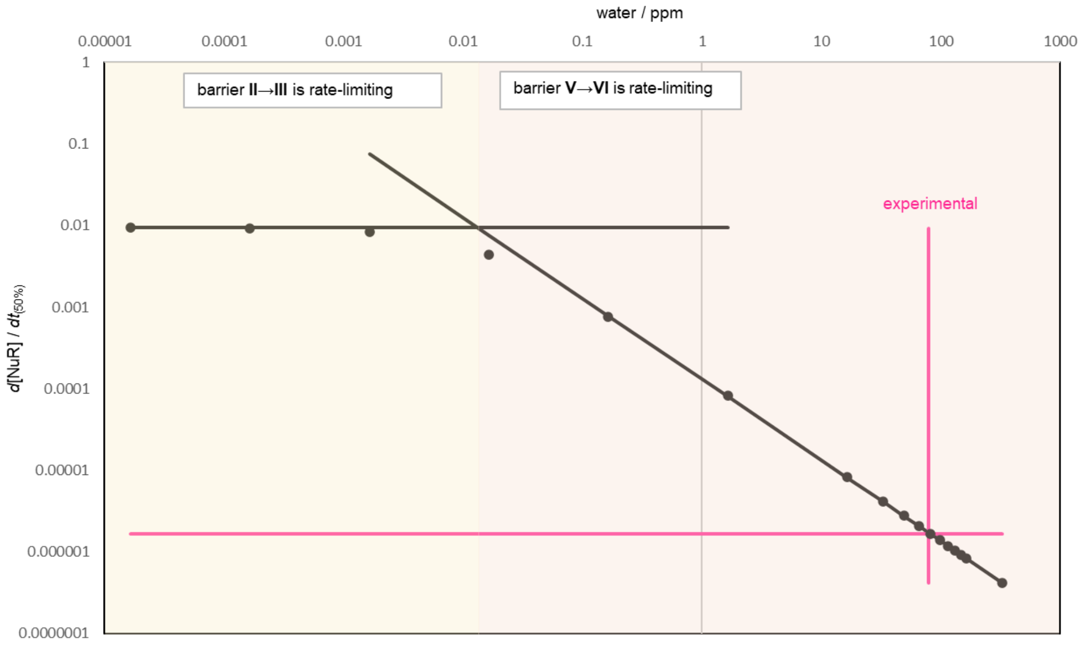

Figure S20. Plot of the rate of formation of product (at 50% conversion) *d*[NuR]/*dt*$_{(50\%)}$ against water concentration in ppm.

For catalyst **1a**, the crossover point for rate-determining step (calculated at 50% conversion) is observed at ~0.013 ppm, where the dehydration barrier (**II**→**III**) becomes rate-limiting (overall rate dominated by the k_2 term). Performing the same analysis for catalyst **2a**, brings the crossover point to approx. 1.2 ppm, which is likely within the regime of experimentally accessible dryness.

We further note that at lower conversion extents, the shape of the graph is the same, but the rate-limiting barrier crossover point is found at marginally higher water concentrations, and vice versa (using **1a**: 0.018 ppm water at 10% conversion; 0.011 ppm water at 65% conversion). The slight difference in the value calculated here (**S5.5**) compared to **S5.4** is due to the different assumptions/terms used in the calculation (i.e., a minor effect due to the alcohol concentration which is ignored in **S5.4**.)

S5.6 Kinetic barrier diagrams

Kinetic barrier diagrams were constructed to describe the reaction (Main Text, Figure 4A). Since the calculated energy profile already included entropic correction factors to account for molecularity,[S2] no further adjustments were made. For simplicity, Intermediates **III**–**V** were grouped together as a single energy level corresponding to the energy of the lowest state (**V**). A barrier was added to account for water removal. In Figure 4A(i), the height of the barrier and subsequent lower energy of the dehydrated steps were set based on Eyring conversion from the simulated rate constants. The modifications in Figure 4A(ii) and 4A(iii) are set at an arbitrary illustrative level.

S6. Experimental demonstration of the effect of water removal rate and extent on the catalytic Mitsunobu reaction rate

S6.1 Dean-Stark trap vs overhead desiccant

Following Denton and co-workers,[S1] toluene (12.5 mL) was added to a 25 mL B14-necked round-bottomed flask containing a 4 × 10 mm magnetic stirrer bar, (±)-1-phenylpropan-2-ol (136 mg, 1.00 mmol), 2,4-dinitrobenzoic acid (212 mg, 1.00 mmol) and (2-hydroxybenzyl)diphenylphosphine oxide (62.2 mg, 0.200 mmol, 20 mol%) and the flask connected to either:

(A) a Dean–Stark apparatus and a condenser open to air via a rubber septum with a needle (0.8 mm bore), or

(B) a glass bulb holding 8.4 g oven dried 3 Å ball-form molecular sieves (held in the bulb with aluminum foil such that refluxing solvent would rapidly contact the sieves and drip back to the reaction), and a condenser open to air via a rubber septum with a needle (0.8 mm bore). We refer to this set-up as the “overhead desiccant” set-up, a variation on a published procedure.[S5]

The reaction was stirred at reflux (oil bath at 130 °C) for 20 h. After this time, an aliquot of the reaction was removed and concentrated, and the conversion to product determined by comparative integration of the acid and ester peaks. A known mass of solvent from the reaction flask (0.5–1 mL) was assessed for water content by Karl–Fischer titration. Three repeat measurements were performed per reaction. This whole process was repeated three times to yield the data in **Table S9**. Note, to push the system further, the initial wetness of the toluene in the overhead desiccant method was artificially increased to ~double that of the Dean–Stark method.

Table S9. Conversion to ester and starting/ending water content during the catalytic Mitsunobu reaction using two drying techniques.

ID	conditions	conversion (%)	H_2O (ppm) start	H_2O (ppm) end
A1	Dean-Stark	8.2	266.8	50.9 (±3.8)
A2	Dean-Stark	12.0	245.5	70.2 (±1.6)
A3	Dean-Stark	10.7	270	136.3 (±1.5)
	**AVERAGE**	10.3	260.8	85.8
	**ERROR**	±2.1	±14.6	±49.1
B1	overhead sieves	15.1	525.7	13.1 (±2.1)
B2	overhead sieves	15.8	523.4	12.0 (±2.1)
B3	overhead sieves	14.4	510.2	8.7 (±1.1)
	**AVERAGE**	15.1	519.8	11.3
	**ERROR**	±0.8	±9.2	±2.5

experiments performed at 0.08 M, 12.5 mL, toluene heated at 130 °C for 20 h
errors quoted as 95% confidence intervals from three repeats

S6.2 Kinetic analysis of experimental data

Having obtained data for the conversion of product, we applied our kinetic model (Section S3) to describe the data, allowing the rate constant for water removal to be fitted to allow the model to fit the data. We use the experimentally determined initial water concentration in the modelling. The final water concentration was used to set the ratio of the rates for the water removal and reintroduction. Fitting in COPASI[S3] through an evolutionary programming method thus allowed the rate constants for the removal/addition of water to be determined to fit the experimentally determined conversion.

A

Process	Reaction	Forward reaction	Rate constant		Backward reaction	Rate constant	
I→II	**1a** + NuH ⇋ **1b**	k_1	7.02 × 10^{12}	s^{-1} M^{-1}	k_{-1}	6.64 × 10^{11}	s^{-1}
II→III	**1b** ⇋ **1c** + H_2O	k_2	6.10 × 10^{0}	s^{-1}	k_{-2}	6.92 × 10^{7}	s^{-1} M^{-1}
III→IV	**1c** + ROH ⇋ **1d**	k_3	7.02 × 10^{12}	s^{-1} M^{-1}	k_{-3}	3.03 × 10^{11}	s^{-1}
IV→V	**1d** ⇋ **1e**	k_4	1.12 × 10^{12}	s^{-1}	k_{-4}	3.93 × 10^{11}	s^{-1}
V→VI	**1e** ⇋ **1a** + RNu	k_5	3.90 × 10^{-1}	s^{-1}	k_{-5}	7.55 × 10^{-8}	s^{-1} M^{-1}
Water removal (Dean–Stark)	H_2O ⇋ H_2O (removed)	k_6	2.20 × 10^{-5}	s^{-1}	k_{-6}	1.16 × 10^{-6}	s^{-1}
Water removal (overhead desiccant)	H_2O ⇋ H_2O (removed)	k_6	6.30 × 10^{-5}	s^{-1}	k_{-6}	3.80 × 10^{-7}	s^{-1}
Uncatalyzed	NuH + ROH ⇋ RNu + H_2O	k_7	1.97 × 10^{-11}	s^{-1} M^{-1}	k_{-7}	6.17 × 10^{-14}	s^{-1} M^{-1}

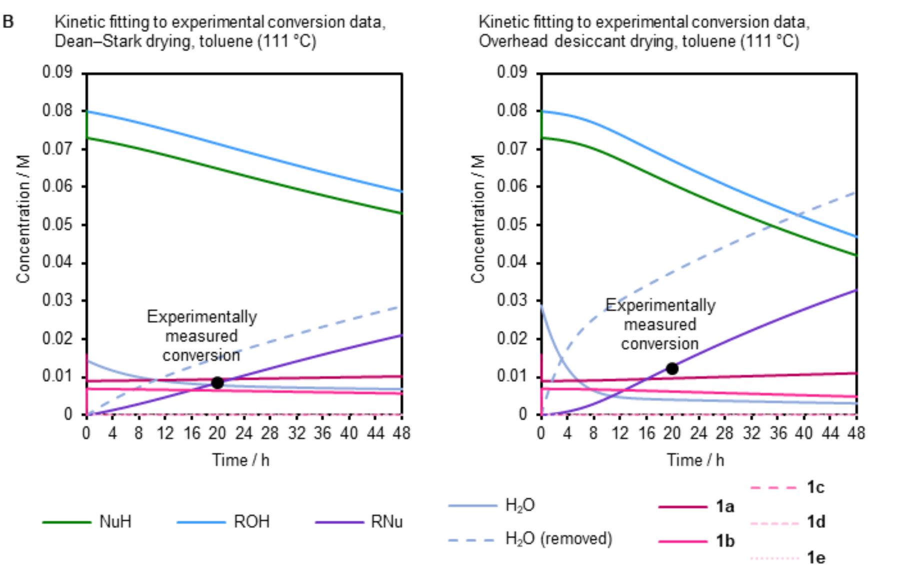

Figure S17. Reaction profiles fitted to experimentally determined conversion data (Section S6.1) for the catalytic Mitsunobu reaction performed in toluene with different drying techniques. (**A**) Table of rate constants. (**B**) Simulated reaction profiles based on reported rate constants.

S7. References

S1. Beddoe, R. H.; Andrews, K. G.; Magné, V.; Cuthbertson, J. D.; Saska, J.; Shannon-Little, A. L.; Shanahan, S. E.; Sneddon, H. F.; Denton, R. M. Redox-neutral organocatalytic Mitsunobu reactions. *Science* **2019**, *365*, 910–914. DOI: 10.1126/science.aax3353

S2. Zou, Y.; Wong, J. J.; Houk, K. N. Computational exploration of a redox-Neutral organocatalytic Mitsunobu reaction. *J. Am. Chem. Soc.* **2020**, *142*, 16403–16408. DOI: 10.1021/jacs.0c07487

S3. Hoops, S.; Sahle, S.; Gauges, R.; Lee, C.; Pahle, J.; Simus, N.; Singhal, M.; Xu, L.; Mendes, P.; Kummer, U. COPASI: a COmplex PAthway SImulator. *Bioinformatics* **2006**, *22*, 3067–3074. DOI: 10.1093/bioinformatics/btl485.

S4. Song, D.; Zhang, C.; Cheng, Y.; Chen, L.; Lin, J.; Zheng, C.; Liu, T.; Ding, Y.; Ling, F.; Zhong, W. Development of a more efficient catalyst for the redox-neutral organocatalytic Mitsunobu reaction by DFT-guided catalyst design. *Green Synth. Catal.* **2024**, 5, 290-296. DOI: 10.1016/j.gresc.2023.11.002.

S5. Eisenbraun, E. J.; Payne, K. W. Dean–Stark apparatus modified for use with molecular sieves. *Ind. Eng. Chem. Res.* **1999**, 38, 4521–4524. DOI: 10.1021/IE9904044
